# Integrated Analysis of Liver Transcriptome, miRNA, and Proteome of Chinese Indigenous Breed Ningxiang Pig in Three Developmental Stages Uncovers Significant miRNA–mRNA–Protein Networks in Lipid Metabolism

**DOI:** 10.3389/fgene.2021.709521

**Published:** 2021-09-16

**Authors:** Biao Li, Jinzeng Yang, Yan Gong, Yu Xiao, Qinghua Zeng, Kang Xu, Yehui Duan, Jianhua He, Jun He, Haiming Ma

**Affiliations:** ^1^College of Animal Science and Technology, Hunan Agricultural University, Changsha, China; ^2^Department of Human Nutrition, Food and Animal Sciences, University of Hawaii at Manoa, Honolulu, HI, United States; ^3^Ningxiang Pig Farm of Dalong Livestock Technology Co., Ltd., Ningxiang, China; ^4^Laboratory of Animal Nutritional Physiology and Metabolic Process, Key Laboratory of Agroecological Processes in Subtropical Region, Institute of Subtropical Agriculture, Chinese Academy of Sciences (CAS), Changsha, China

**Keywords:** Ningxiang pigs, liver development, transcriptome, miRNome, proteome, lipid metabolism

## Abstract

Liver is an important metabolic organ of mammals. During each transitional period of life, liver metabolism is programmed by a complex molecular regulatory system for multiple physiological functions, many pathways of which are regulated by hormones and cytokines, nuclear receptors, and transcription factors. To gain a comprehensive and unbiased molecular understanding of liver growth and development in Ningxiang pigs, we analyzed the mRNA, microRNA (miRNA), and proteomes of the livers of Ningxiang pigs during lactation, nursery, and fattening periods. A total of 22,411 genes (19,653 known mRNAs and 2758 novel mRNAs), 1122 miRNAs (384 known miRNAs and 738 novel miRNAs), and 1123 unique proteins with medium and high abundance were identified by high-throughput sequencing and mass spectrometry. We show that the differences in transcriptional, post-transcriptional, or protein levels were readily identified by comparing different time periods, providing evidence that functional changes that may occur during liver development are widespread. In addition, we found many overlapping differentially expressed genes (DEGs)/differentially expressed miRNAs (DEMs)/differentially expressed proteins (DEPs) related to glycolipid metabolism in any group comparison. These overlapping DEGs/DEMs/DGPs may play an important role in functional transformation during liver development. Short Time-series Expression Miner (STEM) analysis revealed multiple expression patterns of mRNA, miRNA, and protein in the liver. Furthermore, several diverse key Kyoto Encyclopedia of Genes and Genomes (KEGG) pathways, including immune defense, glycolipid metabolism, protein transport and uptake, and cell proliferation and development, were identified by combined analysis of DEGs and DGPs. A number of predicted miRNA–mRNA–protein pairs were found and validated by qRT-PCR and parallel reaction monitoring (PRM) assays. The results provide new and important information about the genetic breeding of Ningxiang pigs, which represents a foundation for further understanding the molecular regulatory mechanisms of dynamic development of liver tissue, functional transformation, and lipid metabolism.

## Introduction

Metabolism of lipid in animals is highly dependent upon liver development and physiology (Liu et al., 2018). The type and content of lipids determine the quality of meat. Data from several mammalian studies suggest that microRNAs (miRNAs) play an important regulatory role in liver lipid metabolism. For example, miR-122, which is highly expressed in mammalian liver, specifically inhibitions the expression of genes involved in cholesterol biosynthesis by targeting the regulation of cationic amino acid transporter 1 (*CPT1*α), thereby regulating liver fatty acid (FA) and cholesterol synthesis (Iliopoulos et al., 2010). One miRNA can regulate the expression of several mRNAs, and the expression of one mRNA can be simultaneously regulated by several miRNAs. Li H. et al. (2016) found that fatty acid desaturase 1 (*FADS1*) was targeted by miR-365-3p, miR-218-5p, miR-181a-5p, miR-181b-5p, miR-29a-3p, and miR-23b-3p, while fatty acid desaturase 2 (*FADS2*) was targeted by miR-30c-1-3p, which was found in the study of differences in miRNA expression in liver of 20- and 30-week-old hens. In addition, it has been reported that fatty acid synthase (*FASN*) and stearoyl-COA desaturease (*SCD*) can be jointly targeted directly by miR-212-5p (Guo et al., 2017) and miR-27a (Zhang M. Y. et al., 2017), affecting the accumulation of triglyceride (TG) in hepatocytes.

In vertebrates, the transformation of liver metabolic program during each transitional period is a complex molecular regulatory system, whether in embryo, postnatal developmental stages and adulthood. For example, the yolk lipids of newly hatched chicks are rapidly depleted and metabolically converted to a carbohydrate-based energy source. Due to a poorly developed digestive system, they cannot process feed as efficiently as adult chickens (Uni et al., 2005). By using delayed feeding for 48 h to prevent liver metabolic transformation, found that delayed feeding resulted in increased miR-20b expression, while its target *FADS1* expression was conversely decreased. It shows that the *FADS1* gene is mediated by miRNA and participates in this transformation before and after transcription (Hicks et al., 2019). At the same time, the expression of miR-33 was significantly reduced, and miR-33, a well-known lipid metabolism regulator in vertebrates, has been proved to inhibit the oxidation of FAs in the liver (Shao et al., 2019). It is suggested that *de novo* synthesis of FAs can be almost inhibited during the hepatic metabolic transition, which may be caused by the decreased expression of *SREBF1* and its downstream *FADS*, and miRNAs may be the key regulatory factors of the hepatic metabolic pathway during this metabolic transition. miRNAs may also play an important role in the prolongation of circulation responses of liver FAs. For example, Zhang M. et al. (2017) demonstrated that miR-218-5p can directly target *ELOVL5* and indirectly promote the synthesis of LCPUFA in the liver, and this regulation may be dependent on miRNAs. Subsequently, it has been found that *ELOVL2* and ultra-long chain PUFA concentrations were downregulated in the dedifferentiated primary human hepatocytes (PHHs). Furthermore, a variety of miRNAs were differentially expressed during the dedifferentiation of PHHs, such as up-regulated miR-27a and down-regulated miR-30, suggesting that these miRNAs are involved in the synthesis, accumulation, and secretion of PHH lipids (Kiamehr et al., 2019).

Ningxiang pig is one of the four most famous native pig breeds in China, with a domestication history of more than 5000 years. Ningxiang pigs, known for their meaty taste, have more than 5% intramuscular fat content, while compared with about 2% for imported commercial pigs (He et al., 2020). However, the molecular mechanism of Ningxiang pig liver regulating FA synthesis and metabolism and other meat quality related traits is poorly understood. FA synthesis and metabolism are affected by many factors such as feed, variety, and environment. In addition, there are many genes, transcripts, and proteins involved in each stage of tissue development of different pig breeds. The single factor molecular research based on the comparison of different pig breeds of the same age or weight cannot fully elucidate the molecular mechanisms and interactions in the organism. Therefore, the study of the dynamic expression of tissue development and the changes of transcription factors are important bases for understanding the transformation of tissue function during the development of pigs. However, due to the limitation of transcriptional regulation, transcriptional changes cannot accurately describe various changes in protein levels (Li et al., 2018). In this case, proteomics can help reveal changes in metabolic pathways in tissues, not only for biomarkers in breeding programs, but also for defining animal models (Bassols et al., 2014; Cui et al., 2016). Label-free liquid chromatography–mass spectrometry (LC–MS) has been increasingly used to quantify protein expression and compare different samples compared to traditional proteomic profiling methods (two-dimensional polyacrylamide gel electrophoresis and mass spectrometry). This approach may also offer the possibility of better studying low molecular weight or high molecular weight hydrophobic proteins, which are very challenging for analysis using traditional proteomic methods (Neilson et al., 2011). Bovo et al. (2018) used label-free LC–MS proteomics to compare the liver proteomic characteristics of Italian Duroc and Italian Great White pigs, and analyzed a total of 25 proteins that clearly distinguish Italian Duroc and Italian Great White pigs.

It has been reported that the scalability of protein studies techniques are not as good as nucleic acid (Manzoni et al., 2018). For this purpose, we combined analysis of mRNA-seq, miRNA-seq, and proteomics in liver from Ningxiang pigs at different ages (30, 90, and 210 days after birth), which is the first report on the integrated analysis of Ningxiang pig liver, also validated by qRT-PCR and parallel reaction monitoring (PRM) assays (Peterson et al., 2012). Our data suggest that the potential miRNA regulation of gene expression and the regulatory mechanism of protein expression serve as the basis for future research on pork quality traits in Ningxiang. The ultimate goal is to identify the key dynamic regulatory networks of liver on lipid metabolism and FA synthesis during the growth and development of Ningxiang pigs, and then to inform the biological mechanisms that may affect their performance.

## Results

### Morphological Characteristics of Liver of Ningxiang Pigs at Different Stages

**FIGURE 1:**
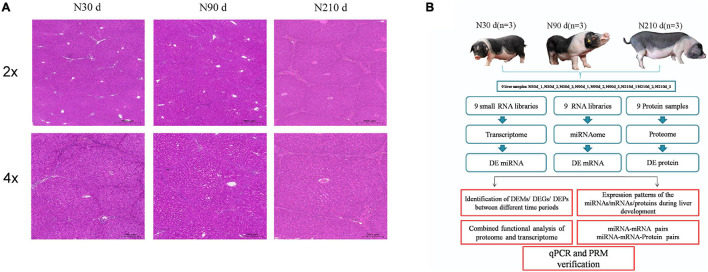
Hematoxylin and eosin staining of spleens and overall study design. **(A)** H&E staining results of spleens in three developmental stages of Ningxiang pigs. **(B)** Schematic workflow of the experimental design of this study.

In order to observe the morphological differences of the liver of Ningxiang pigs during the important period of postnatal growth and development. Liver samples from Ningxiang pigs at the same location were collected at 30, 90, and 210 days of age, respectively. These time points were determined according to the growth characteristics of Ningxiang pigs. Hematoxylin and eosin (H&E) staining showed a gradual increase in connective tissue in the liver at 90 and 210 days of age, and a gradual increase in hepatic lobular sepsis, compared with 30 days of age. Especially at 210 days of age, the hepatic lobules were significantly enlarged and the liver was mature (Figure 1A). These results indicate that a series of biological processes take place in the liver of Ningxiang pigs after birth. Therefore, we combined multiple omics sequencing analyses to systematically study the roles of miRNA, mRNA, and protein in the biological processes of different stages of liver development (Figure 1B).

### Summary of Transcriptome, miRNAome, and Proteome

**FIGURE 2:**
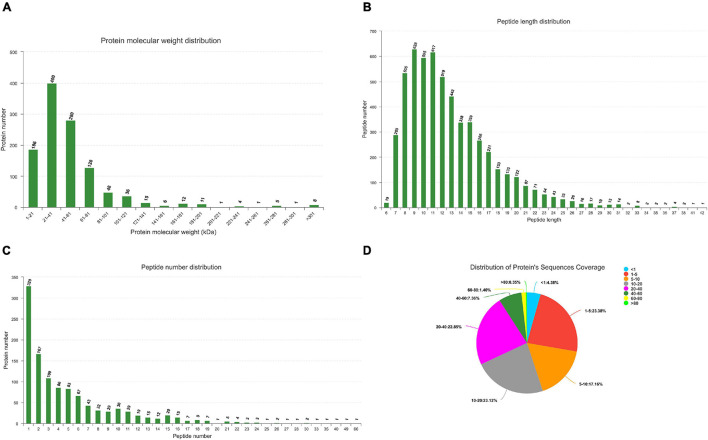
Identification and feature of proteins in Ningxiang pigs. **(A)** Distribution of protein molecular weight. **(B)** Length distribution of peptide. **(C)** Number distribution of peptide. **(D)** Distribution of protein’s sequnences coverage. Each sector represents the proportion of a coverage range. The number outside the sector represents the coverage range and the proportion of proteins distributed in this range.

All MS/MS spectra were processed using Proteome Discoverer software. Label free analysis of Ningxiang pig liver proteome showed 367,412 spectra in this database and resulted in 5624 identified peptides. In summary, a total of 22,411 unique genes (including 19,653 known mRNAs and 2758 novel mRNAs) and 1122 unique miRNAs (including 384 known miRNAs and 738 novel miRNAs) had been identified (Supplementary Files 1, 2). The quality control analysis of miRNAome can be found in Supplementary File 3. Peptide/protein identification and quantification of all proteins are shown in Supplementary File 4. Protein molecular weight distribution diagram, peptide length distribution diagram, specific peptide number distribution diagram, and protein sequences coverage are shown in Figure 2.

### Screening and Functional Analysis of Differentially Expressed Proteins

**FIGURE 3:**
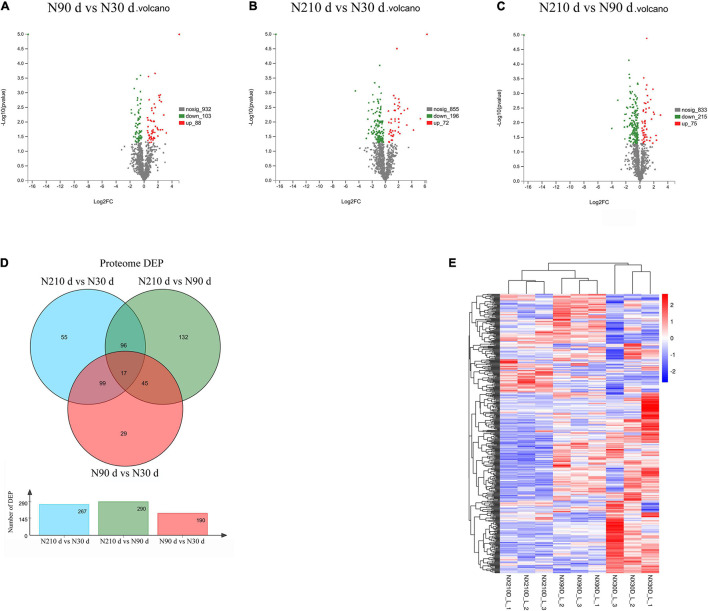
Overview of the differentially expressed proteins. **(A–C)** Differentially expressed protein between different growth stages in postnatal liver. **(D)** The Venn diagram of DEPs at three comparison groups. **(E)** Heatmap of proteins expression abundance at 30, 90, and 210 days.

**FIGURE 4:**
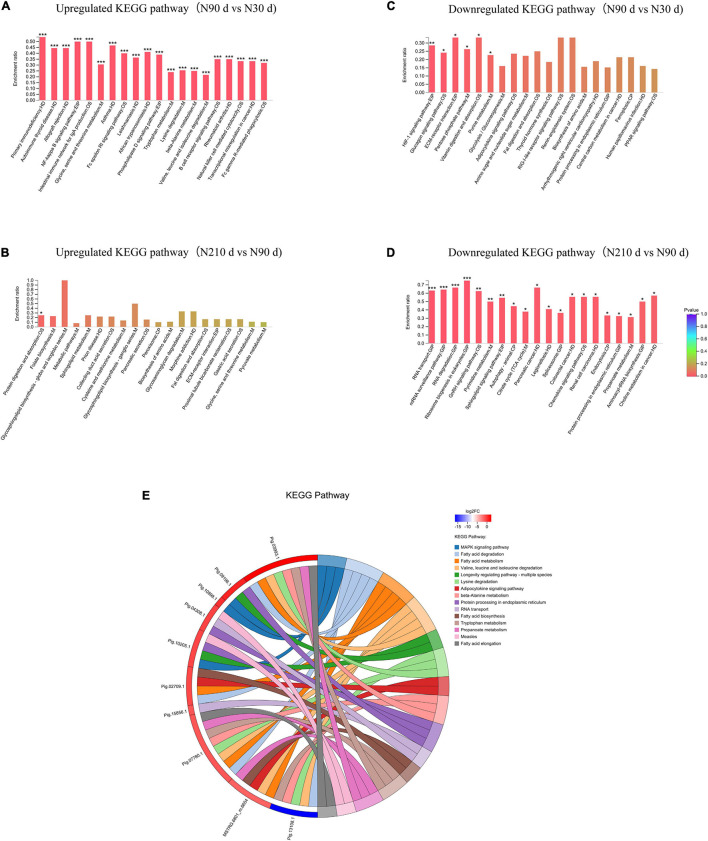
Kyoto Encyclopedia of Genes and Genomes analysis of DEPs in the N90 vs. N30 d and N210 vs. N90 d groups. **(A)** Upregulated KEGG analysis of DE proteins in the N90 vs. N30 d group. **(B)** Upregulated KEGG analysis of DE proteins in the N210 vs. N90 d group. **(C)** Downregulated KEGG analysis of DE proteins in the N90 vs. N30 d group. **(D)** Downregulated KEGG analysis of DE proteins in the N210 vs. N90 d group. The top 20 enriched KEGG pathways ranked by *P*-values are shown. *P < 0.05, **P < 0.01, ***P < 0.001. **(E)** Chord diagram of overlapped DEPs enrichment.

A total of 753 differentially expressed proteins (DEPs) were identified in the three groups (Figures 3A–C): 192 DEPs were identified at N90 vs. N30 d, of which 88 were upregulated and 104 were downregulated. N210 vs. N30 d had 268 DEPs, including 72 upregulated DEPs and 196 downregulated DEPs. N210 vs. N90 d had 293 DEPs, among which 75 were up-regulated and 215 were down-regulated. Based on the analysis of the number of DEPs in the three groups, it was found that the number of DEPs in the N90 vs. N30 d group was far less than that in the N210 vs. N90 d group and the N210 vs. N30 d group. The number of DEPs in N210 vs. N90 d and N210 vs. N30 d groups showed the same trend, and most DEPs were decreased at N210 and N90 d. In addition, we identified 29, 55, and 132 DEPs that were uniquely differentially expressed in the N90 vs. N30 d group, N210 vs. N30 d group, and N210 vs. N90 d group, respectively, and 17 DEPs were co-expressed in the three groups (Figure 3D). The protein expression of DEPS in liver tissues of Ningxiang pigs of different ages was analyzed by hierarchical clustering method (Figure 3E). Three biological replicas in each age group showed similar expression trends. In addition, N90 and N210 d DEPs showed similar expression patterns, which indicated that N30 to N90 d was the key stage for the liver growth and development of Ningxiang pigs. In order to further understand the DEPs in three key growth stages involved in regulating the liver function, only the adjacent time point to compare differences in protein Kyoto Encyclopedia of Genes and Genomes (KEGG) enrichment, we found that compared with 30 days, the 90 days upregulated protein significantly enriched in the 63 regulation pathway, covers almost all of the liver function (Figure 4A). For example, Primary immunodeficiency, Autoimmune thyroid disease, Allograft rejection, and NF-kappa B signaling pathway related to immune defense are extremely significant enrichment. The metabolic pathways related to Glycine, serine, and threonine metabolism, Tryptophan metabolism, Lysine degradation, Metabolism of xenobiotics by cytochrome P450 and FA degradation were significantly enriched. However, compared with 90 days, the 210 days upregulated proteins were only significantly enriched in Protein and absorption (Figure 4B). At the same time, The downregulated DEPs in the N90 vs. N30 d group were enriched only in HIF-1 signaling pathway, Glucagon signaling pathway, ECM-receptor interaction, and Pentose phosphate pathway-related pathways that control cell proliferation and differentiation and maintain cell glucose homeostasis (Figure 4C). However, the number of downregulated pathways in N210 vs. N90 d group was much more than that in N90 vs. N30 d group, which were mainly involved in the control of RNA transcription and protein translation, which indicated that the development of hepatocytes slowed down (Figure 4D and Supplementary File 5). The results showed that the main stage of liver development was from 30 to 90 days of age, during which the liver function might have been gradually matured. Next, in the KEGG enrichment pathway of liver overlapping DEPs, only 14 pathways were significantly identified (*P* < 0.05, Figure 4E). Among these pathways, lipid metabolism and amino acid metabolism are more abundant, among which proteins Pig.02709.1, Pig.07780.1, Pig.03993.1, and MSTRG.6801_M.6654 are involved in the synthesis and elongation of FAs. Their protein-coding genes are Acyl-CoA synthetase long chain family member 1 (*ACSL1*), methylglutaconyl-CoA hydratase (*AUH*), and ethylmalonyl-CoA decarboxylase (*ECHDC1*), respectively. Although the number and function of overlapping DEPs protein enrichment pathways identified are far less than that of differentially expressed genes (DEGs) (Supplementary File 6), these overlapped co-expressed differential proteins play an irreplaceable role in FA synthesis and metabolic balance during liver development in Ningxiang pigs.

**FIGURE 5:**
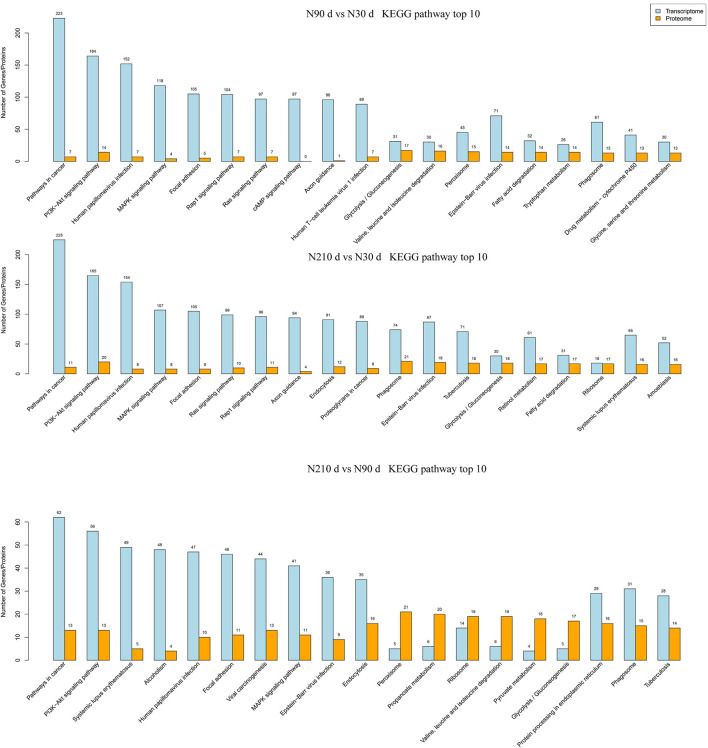
The number of DEG and DEP enriched in KEGG pathway was analyzed by combined transcriptome and protein analysis.

Furthermore, the KEGG pathway enrichment analysis was performed for the combination of different genes and proteins in each group (Figure 5). We found that these differential genes and proteins are mainly concentrated in immune defense, antioxidant capacity, metabolism (amino acids, lipids, and carbohydrates), and protein processing. However, the number of differentially enriched genes in pathways between 90 and 210 days of age was far less than that of 90 vs. 30 d and 210 vs. 30 d. But the number of differentially enriched proteins did not change much, suggesting that gene regulation may be more active in the liver at 30 days of age.

### Liver Proteome Combined Analysis With miRNA and Transcriptome

**FIGURE 6:**
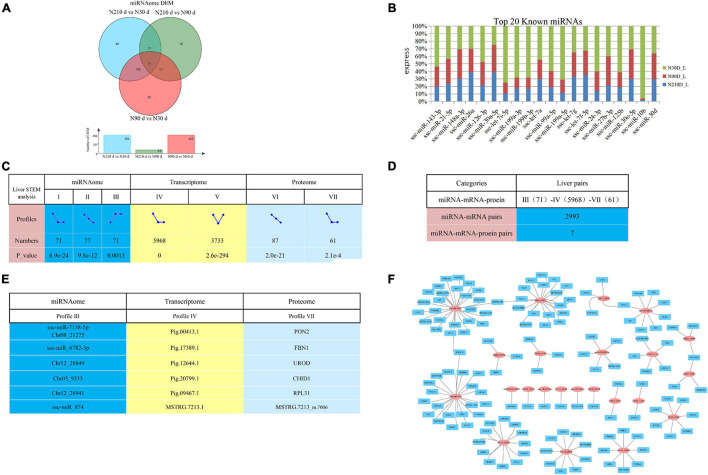
Overview of possible negative miRNA–mRNA–protein interactions. **(A)** The Venn diagram of DEMs at three comparison groups. **(B)** Top 20 known miRNAs expressed in liver. **(C)** STEM analysis of miRNA, transcriptome, and proteome. And count the number and *P*-value of miRNA/mRNA/protein enriched in each profile. **(D)** The numbers of possible negative miRNA–mRNA/miRNA–mRNA–protein interactions based on STEM analysis expression patterns. **(E)** The information of seven potential miRNA–mRNA–protein interaction pairs based on STEM analysis. **(F)** Part of the miRNA–mRNA negative correlation network based on 2993 profile (III)–profile (IV) expression patterns. Red circles indicate miRNAs, Blue rectangle indicate coding genes.

MicroRNAs are important epigenetic factors that bind to mRNA to inhibit post transcriptional gene expression in eukaryotes. By comparing any two groups, a total of 556 differentially expressed miRNAs (DEMs) were identified, of which 50, 48, and 15 DEMs were uniquely differentially expressed in N30 vs. N90 d group, N30 vs. N210 d group, and N90 vs. N210 d group, respectively. Eleven DEMs were co-expressed in the three groups (Figure 6A). We also found high expression of some miRNAs known to be involved in liver development and FA metabolism, such as ssc-miR-143-3p, ssc-miR-21-5p, and ssc-miR-148a-3p, etc. (Figure 6B).

Correlation analysis of miRNA, mRNA, and protein expression data can effectively identify and evaluate the function of miRNA. In order to explore the dynamic expression patterns of Transcriptomic, miRNA, and Proteomic, we adopted the Short Time-series Expression Miner (STEM) analysis method. We found that Transcriptomic, miRNA, and Proteomic had multiple significantly enriched pattern profiles in the three stages of liver development (Figure 6C, *P*-value < 0.05). Surprisingly, Time series analysis showed that Transcriptomic, miRNA, and Proteomic were dynamically expressed during liver development, with may be dynamically correlated expression patterns. This implies that there is a functional correlation or regulatory relationship between Transcriptomic, miRNA, and Proteomic. Take into account miRNAs negatively regulate the expression of their target mRNA through target RNA cleavage, it is generally believed that the expression pattern of miRNA target genes is negatively correlated with the expression pattern of miRNAs. In consideration of the three time points (30, 90, and 210 days), we conducted negative regulation analysis in expression patterns to dissect the possible miRNA–mRNA or miRNA–mRNA–protein interactions. We found that profile III and profile IV presented the opposite expression pattern, which may have a negative regulatory relationship. In order to predict the genes targeted by miRNAs, two softwares (miRanda 3.3a and RNAhybrida) were simultaneously used to identify miRNA binding sites. Finally, combine the data predicted by the two algorithms and calculate the overlaps. We found that there were 2993 negative miRNA–mRNA interaction pairs (Figure 6D and Supplementary File 7) in profile III (71)–profile IV (5968), involving 54 miRNAs (including 31 newly identified miRNAs) and 2138 mRNAs. And part of the miRNA–mRNA interactions network is shown in Figure 6F. Combined with STEM analysis of the proteome, we found a total of seven negative miRNA–mRNA–protein interaction pairs (Figure 6E).

**FIGURE 7:**
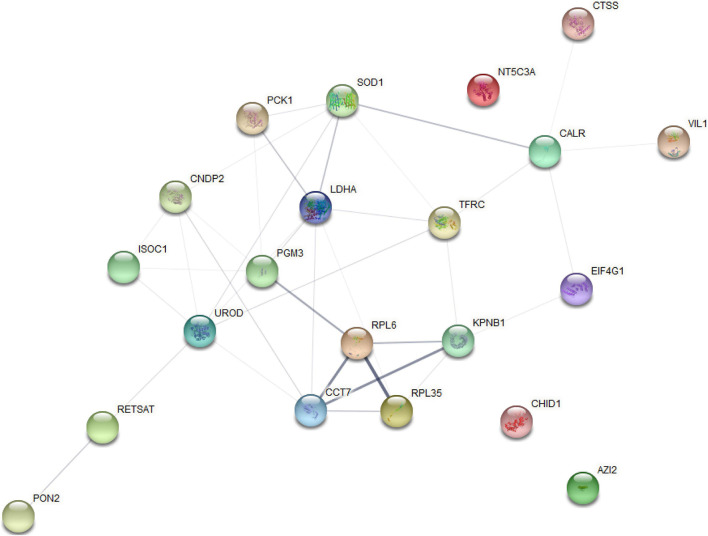
Protein–protein interaction network analysis based on the profile (VII) for STEM analysis.

The analysis of significantly enriched model profile VII revealed a connected protein network (Figure 7 and Supplementary File 8) divided in: (1) one big module composed by 18 nodes and 36 links, (2) a small component of three singletons. In this network most of the proteins interacted with multiple other partners. However, we found that the proteins uroporphyrinogen decarboxylase (*UROD*) and lactate dehydrogenase A (*LDHA*) have the highest degree of connectivity (seven edges), which may enable them to a role as “hub” proteins playing controllers inside biochemical pathways.

### Data Verification of DEMs, DEGs, and DEPs

**FIGURE 8:**
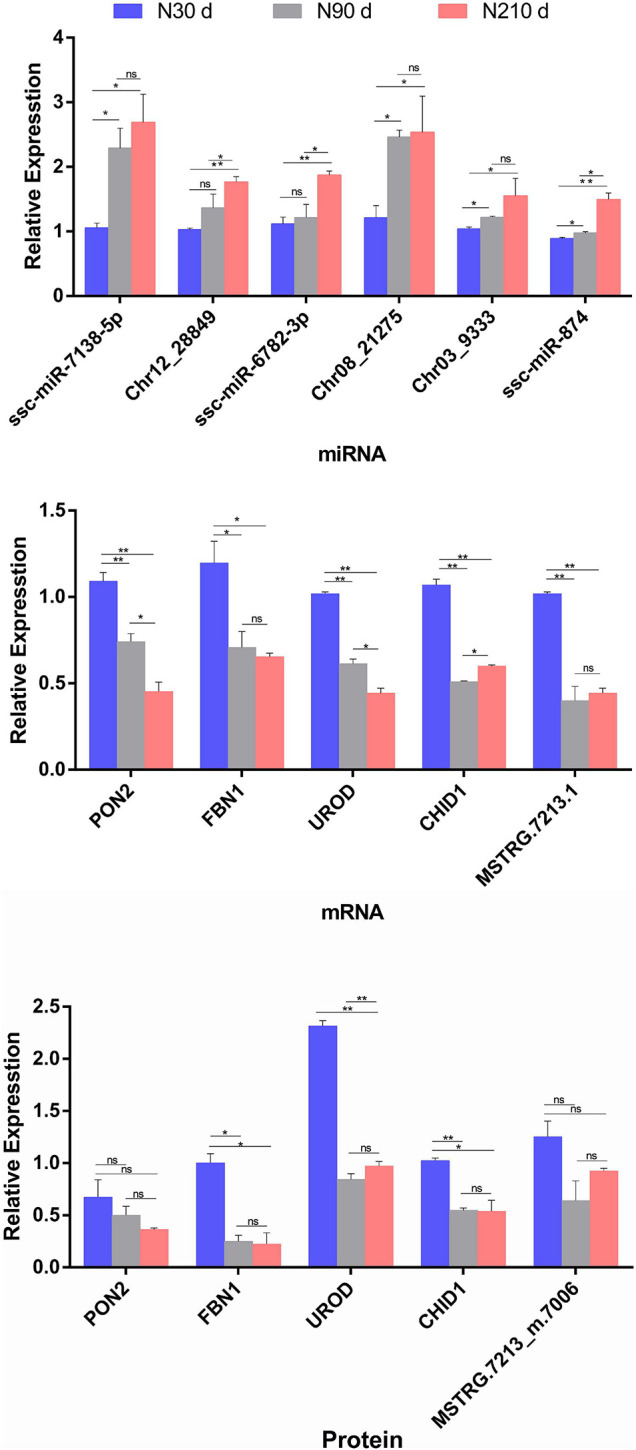
Relative miRNA/mRNA/protein expression of seven selected miRNA–mRNA–protein interaction pairs for comparison of three time periods (30, 90, and 210 days), in respect to miRNAome/transcriptome and qRT-PCR, or proteome and parallel reaction monitoring (PRM). The histograms represent the result of qRT-PCR/PRM verification. Data are represented as mean ± SEM, *n* = 3 per group. *P < 0.05, **P < 0.01.

Although these data in this study have been subjected to rigorous statistical and bioinformatics analysis, three biological duplicates were analyzed to eliminate most possible errors. However, we adopted qRT-PCR and PRM method analysis on selected DEMs/DEGs/DEPs to verify the mRNA-seq/miRNA-seq/label-free sequencing results (Figure 8). These miRNAs/mRNAs/proteins were artificially selected as representatives for their possible roles in lipid deposition and FA synthesis. These were found to be correlated with liver lipid metabolism [ssc-miR-7138-5p-*PON2* (Fuhrman, 2012; Strakovsky et al., 2014) and Chr12_28849-*UROD* (Fader et al., 2017)] and liver glycogen catabolism [ssc-miR-6782-3p-*FBN1* (Bhadel et al., 2020; Yuan et al., 2020)], disease, and inflammatory immunity [Chr03_9333-*CHID1* (Kovaleva et al., 2021)] according to the possible negative miRNA–mRNA–protein interactions on the basis of the STEM analysis during liver development (30–90–210 days) expression patterns.

The PRM method was used to analyze 15 target proteins from liver samples in three time periods. All the second-order mass spectrum matching figures of the target protein candidate peptides can be found in Supplementary Figure 1. See Supplementary File 9 for all the raw data of Panorama. The data analyses of PRM quantitative Skyline in the target peptide section can be found in Supplementary Figure 2. The results show that quantitative data of target peptide fragments could be obtained from all the samples (three replicates at 30, 90, and 210 days, respectively). The results of qRT-PCR/PRM revealed that the most selected miRNAs/mRNAs/proteins have similar expression tendencies with those from mRNA-seq/miRNA-seq/label-free data when compared with 30 days (*P* < 0.05).

In addition, 11 negative DE miRNA/DE mRNA involved in key biological processes such as growth and development, glucose metabolism, and FA synthesis, were selected from the 90 vs. 30 d, 90 vs. 210 d, and 210 vs. 30 d groups, respectively, and quantified by qRT-PCR to verify the transcriptome results (Supplementary Figure 3). For example, the synthesis of unsaturated FAs [Chr10_25757-*FADS3* and ssc-miR-671-5p-*ECHDC1* (Linster et al., 2011; Dewulf et al., 2019)], FA transport [ssc-miR-874-*SLC27A3* and Chr03_9333-*SLC27A1* (Gallardo et al., 2013)], and FA metabolism [ssc-miR-4332-*UCP* (Argentato et al., 2018) and ssc-miR-885-3p-*CCN4* (Tacke et al., 2018)]. Meanwhile, PRM data were combined to verify the changes of key proteins involved in FA synthesis in the proteome, and serve as Supplementary Figure 4 in the discussion. Even if there are some quantitative differences between these analytical methods, the similarity of the expression tendencies between RNA-seq/miRNA-seq/label-free data and qRT-PCR/PRM indicates that these data is reproducible and reliable.

## Discussion

Depending on species and age, the transformation of the hepatic metabolic program during each transitional period of life is a complex molecular regulatory system, many of which are regulated by nuclear receptors and transcription factors, as well as by other dietary and hormonal factors (e.g., carbohydrates, alcohol, insulin). Despite the complexity and degree of coordination of liver lipid mechanisms, any metabolite spillover or nuclear receptor changes will lead to impaired organ function and subsequent pathological changes (Nguyen et al., 2008; Hodson and Gunn, 2020). However, Ningxiang pigs, as a fatty breed, not only have the reputation of excellent meat quality, but also have higher levels of alanine aminotransferase (ALT), ascorbic aminotransferase (AST), and lactate dehydrogenase (LDH-L) in blood than European breeds (Zhao et al., 2012). The liver can be considered a key organ for translating growth rate, feed efficiency, and performance potentials of the animals by exploiting tissue specific metabolic functions. Therefore, the liver of Ningxiang pigs at different growth and development stages is a good model for the study of liver function transformation and lipid metabolism. But because of the influence of the long-term foreign pig breeds, Ningxiang pig population was reduced, especially pure Ningxiang pig were much rarer, and therefore limited understanding of the genetic background of Ningxiang pig. Our current results include detailed information on parallel mRNA, miRNA, and protein expression levels in the liver of pured Ningxiang pigs during the important growth and development periods of lactation (30 days of age), nursery (90 days of age), and fattening (210 days of age).

We identified the expression of 10, 218 DE genes in N90 vs. N30 d, N210 vs. N30 d, and N90 vs. N210 d groups from Ningxiang pig livers. Most DE genes in the three groups were enriched in cancer and diseases (Figure 4). Although “Cancer” does not represent physiological and biochemical reactions for pig, the presence of more differentiated genes from the side was associated with cell proliferation and development (Chen et al., 2017). Notably, the “Rap1signaling pathway,” “PI3K-Akt signaling pathway,” and “MAPK signaling pathway” may provide clues to transboundary regulatory genes involved in signal transduction, signaling molecules, and interactions of cell proliferation and development. Some genes associated with cancer, immune diseases, and cell development have also been found in other animal livers (Li Y. et al., 2016; Zhang et al., 2019). However, there was no complete correlation between the number of DEGs and proteins. For example, most DE proteins in N90 vs. N30 d and N210 vs. N30 d groups were enriched in glucose metabolism, amino acid metabolism, and FA metabolism pathways, which was consistent with the main functions of liver. In addition, through KEGG pathway analysis of liver overlapping proteins, 17 key proteins were identified, among which Pig.02709.1 and MSTRG.6801_M.6654 were involved in FA synthesis, and Pig.07780.1 and Pig.03993.1 were involved in FA elongation. These key overlapping DE genes/proteins may play an irreplaceable role in the functional transformation during the growth and development stage.

The development of mammalian tissues is regulated by various miRNAs. However, many of the miRNAs involved remain unknown. In this study, miRNAs in Ningxiang pig livers from weaning piglets to fattening stage were compared and analyzed by high-throughput sequencing. In total, 384 known miRNAs and 738 new miRNAs were identified and predicted in developing pig livers. To our knowledge, this is the first complete report to systematically identify miRNAs during liver development in Ningxiang pigs using RNA-seq data. Therefore, our findings will enrich the understanding of miRNAs related to FA metabolism, glucose metabolism, and amino acid metabolism in Ningxiang pig liver. The expression level of miRNAs showed obvious temporal dynamic characteristics. In this study, 11 DE miRNAs were coexpressed at three developmental stages. In addition, some miRNAs are expressed at higher levels during liver development. This study presented many known ssc-miR-143-3p, ssc-miR-21-5p, ssc-miR-148a-3p, and many new miRNAs (such as Chr07_18810, Chr07_18928, Chr03_8336, etc.), were abundant miRNAs expressed in the liver of Ningxiang pigs at different time points (Supplementary File 1). It has been reported that exogenous miRNA-143-3p can target the 3′UTR of *ANGPTL 8* and regulate the level of high-density lipoprotein cholesterol in hepatocytes (DiStefano, 2019). In addition, miR-21-5p and miR-148a-3p target *PDCD4* and *ERB 3*, respectively, and are involved in regulating liver cell viability and apoptosis (Xiong et al., 2020; Zhang et al., 2020). These identified miRNAs with high expression may play a crucial role in liver development.

As the most widely distributed mammalian FABP, liver fatty acid-binding protein (*FABP 1*) is highly expressed in the most active liver tissues of long-chain fatty acids (LCFAs) (Jiang et al., 2006). It has been reported that *FABP 1* expression is increased during the proliferation and early differentiation of porcine mesenchymal vascular cells, and also regulates the growth and differentiation of embryonic stem cells (Schroeder et al., 2001). Meanwhile, *FABP 1* is required for liver uptake and oxidation of LCFAs, and it interacts with PPARA, which regulates FA catabolism (Erol et al., 2004; Landrier et al., 2004). *FABP 1* may be responsible for the transport and targeting of FAs to catabolic sites during proliferative interstitial – vascular cell and early adipocyte differentiation. In this study, *FABP 1* was significantly higher at 30 days of age than at 90 and 210 days of age, suggesting that *FABP 1* plays an important role in liver tissue development and FA metabolism of Ningxiang pigs during lactation. In addition, many other DEPs related to FA metabolism and fat deposition are down-regulated during liver tissue development, such as *CD36*, *HSPA5*, *FBP*, *ACSL1*, *LDH*, *APMAP*, *ACADM*, etc. FA translocase (*CD36*) is a single chain transmembrane glycoprotein with a special neck ring structure, which is expressed in a variety of tissues of mammals (Spitsberg et al., 1995; Febbraio et al., 2001). *CD36* has been reported to play an important role in adipocyte development and fat deposition, and also act as a receptor for LCFAs, promoting the entry of FAs into cells, thereby promoting the utilization and storage of FAs (Silverstein and Febbraio, 2009; Christiaens et al., 2012). Medium-chain acyl-coenzyme A dehydrogenase (*ACADM*) is a member of the acyl-coenzyme A dehydrogenase (*ACAD*) family, which is mainly involved in the β-oxidation process of medium-chain FAs and amino acids (Kim and Miura, 2004). Lack of *ACADM* hinders the oxidation of FAs and leads to disease or death in mice. This indicates the importance of *ACADM* in FA metabolism (Smith et al., 2010). *APMAP* is a 46 kDa glycosylated type II transmembrane protein with an N-terminal anchor and a six-leaf structure of β-an extracellular domain with potential hydrolase activity and calcium binding (Albrektsen et al., 2001; Bogner-Strauss et al., 2010). *APMAP* is highly upregulated during adipogenic differentiation in various mouse and human cell lines, which is important for *in vitro* adipogenesis, and the absence of Apmap has a protective effect on diet-induced insulin resistance in mice and shows an overall improvement in glucose utilization in high-fat diets (Pessentheiner et al., 2017). It is speculated that these proteins regulate the synthesis and oxidation of FAs by down-regulating their expression during the growth of Ningxiang pig liver. In addition, many other proteins were significantly up-regulated, such as *CD26*, *AUH*, *CDH1*, *EchDDC1*, *EchDDC2*, *HSPB1*, and *PON2*, etc. Cadherin-1 (*CDH1*) is one of two adaptor proteins of the E3 ubiquitin ligase (APC/C) that controls mitosis and DNA replication. So far, the function of CDH1 *in vivo* has not been fully studied. However, it has been found that *CDH1* may be related to the regulation of liver cell cycle. The deletion of *CDH1* will upregulate *Cyclin D1*, lead to the prolongation of S phase and increase DNA replication (Cheng et al., 2017). Weight gain, increased fat mass, and hepatic fat deposition were also observed in *CDH1*-overexpressing mice. These up-regulated proteins may be closely related to the growth and development of liver cells (Baumeier et al., 2017).

At the same time, we found that the proteins *AUH* (Nakagawa et al., 1995), *ECHDC1*, and *ACSL1* were differentially expressed in any two groups of liver comparison. *ACSL1* is a member of the five known acyl-CoA synthases (*ACSLs*). Specific loss of *ACSL1* in the liver reduces triglyceride synthesis and β-oxidation of LCFAs. And changes the composition of phospholipid FAs, while overexpression of *ACSL1* can increase the content of TG in liver and blood, thus indirectly affecting meat quality (Parkes et al., 2006). It is well known that most FAs are straight-chain FAs synthesized by *FASN* using acetyl-CoA and malonyl CoA. However, the absence of *ECHDC1* in adipocytes restricts the accumulation of cytoplasmic methyl and ethylmalonyl-coenzyme A, leading to a significant reduction in the synthesis of linear FAs, indicating that *ECHDC1* may serve as a key modulator of methyl branched FA expression (Dewulf et al., 2019). These key overlapping DE proteins may play an irreplaceable role in the functional transformation during growth and development.

Time series analysis showed that protein-coding genes and miRNAs were expressed dynamically during liver development, with opposite expression patterns. These results indicate that this implies a functional correlation or regulatory relationship between Transcriptomic and miRNA. Since miRNA negatively regulates the expression of its target mRNA through cleavage of target RNA, which provides a basis for protein changes to some extent, the expression pattern of miRNA target genes is generally negatively correlated with the expression pattern of miRNA. For example, ssc-miR-4332-*UCP3*, Chr08_21275-*ACACA*, ssc-miR-885-3p-*CCN4*, ssc-miR-4332-*ATP2A3*, ssc-miR-874-*LDLRAD4*, ssc-miR-138-*LDLRAD3*, and other miRNA–mRNA pairs related to lipid anabolism are in line with the negative regulation law of STEM analysis, which can provide ideas for future research on liver function. From a biological point of view, genes/proteins with similar expression patterns may have common characteristics, such as being regulated by a certain gene at the same time, having similar biological functions, or having a common cellular origin. By STEM analysis, we also observed similar expression trends between mRNAs (profile IV) and proteins (profile VII). Then, for the functional connections established between these proteins with similar expression trends, we found that most of the proteins in this network have multiple other partners interacting with each other. In addition, the protein *UROD* presented the highest degree of connection (seven edges), which may assign to them a role as “hub” proteins playing a putative function of controllers inside biochemical pathways. It has been reported that *UROD* is a cytoplasmic enzyme, which is related to heme production. Heme is essential for all cells and involved in the oxidation of a variety of chemicals, including drugs, vitamins, FAs, and prostaglandins (Bonkowsky et al., 1979; Shimizu et al., 2019). Furthermore, proteins related to lipid metabolism, such as superoxide dismutase 1 (*SOD1*), *LDHA*, and paraoxonase 2 (*PON2*), were also included in this network. Combined with miRNAome data, we found seven negative miRNA–mRNA–protein interactions pairs (including protein *UROD* and *PON2*) from profile III–profile IV–profile VII, indicating that these proteins with similar expression profiles may be regulated by certain genes and participate in the same regulatory pathway. These interaction networks are expected to lay a foundation for the future study of the molecular regulation mechanism of lipid metabolism in Ningxiang pig liver at different periods.

## Conclusion

In this study, RNA-seq, miRNA-seq, label-Free, and PRM were used to analyze the transcriptome and proteome of liver tissues at three key developmental time points (lactation period, nursery period, and fattening period) in Ningxiang pigs. The key dynamic regulatory network related to liver development and FA synthesis and metabolism of Ningxiang pigs was identified. Taken together, liver function is closely related to metabolic and digestive diseases of animals and meat quality traits of livestock. although this study cannot fully explain all the molecular mechanisms in regulation of lipid metabolism in pig liver. Research results from transcription, miRNA, and proteome analysis provide initial database in elucidating the genetic mechanisms underlying diseases and meat quality traits in livestock. It also provides a theoretical basis for future breeding applications.

## Materials and Methods

### Experimental Animals and Sampling

Nine purebred male Ningxiang pigs were selected for this experiment, including 30 days (30 days after birth, *n* = 3), 90 days (90 days after birth, *n* = 3), and 210 days (210 days after birth, *n* = 3). Three groups of pig are half-sibs, and the duplicate samples are full-sibs. According to standard procedures, three healthy Ningxiang pigs of similar weight were selected from three age groups. The pigs are provided by the Ningxiang Pig Farm of Hunan Dalong Animal Husbandry Technology Co., Ltd. Under standard environmental conditions, they were fed three times routinely with free drinking water. Samples of the left liver lobules were collected within 30 min after slaughter, frozen in liquid nitrogen, and stored at −80°C. These harvested samples were used to conduct mRNA-seq, miRNA-seq, proteomics, qRT-PCR, and PRM, respectively. All the above experiments including the mass spectrometry experiments had three biological replicates (N30d_1, N30d_2, N30d_3, N90d_1,N90d_2, N90d_3, N210d_1, N210d_2, and N210d_3).

### Hematoxylin–Eosin Staining

The liver tissue is taken out from the fixative, and the dehydration machine is dehydrated with gradient alcohol. Melt the paraffin at 65° for 3 h. Embed the wax-soaked liver tissue in the embedding machine. The wax block is sliced in a paraffin microtome with a thickness of 4 μm. After roasting the wax, take it out at room temperature and store it for later use. Stain with hematoxylin for 3–5 min, wash with water. The sections were dehydrated with 85 and 95% gradient alcohol in sequence, and stained with eosin staining solution for 5 min. Seal the slices with neutral gum. Microscope inspection, Case Viewer 2.4 image analysis software collects and analyses pictures.

### RNA Isolation, Library Preparation, and Transcriptome Sequencing

According to the TruSeq^TM^ strand total RNA kit from Illumina (San Diego, CA, United States), 5 μg total RNA was used to prepare an RNA-seq transcriptome strand library. After that, the ribosomal RNA (rRNA) was removed using the Ribo-Zero Magnetic kit. And fragmented by fragmentation buffer firstly. Second, random hexamer primers were used to synthesize first-strand complementary DNA (cDNA). Then the RNA template was removed and a replacement strand was synthesized, and dUTP was used instead of dTTP to generate ds cDNA. AMPure XP beads were used to separate ds cDNA from the second-strand reaction mix. An “A” nucleotide was added to the 3′ ends to prevent them from ligating to one another during the adapter ligation reaction. Finally, multiple indexing adaptors were ligated to the ends of the ds cDNA (Cui et al., 2018). After quantification by TBS380, Shanghai Majorbio Bio-Pharm Biotechnology Co., Ltd. (Shanghai, China) provides sequencing technical support.

### Transcripts Assembly

The quality control of raw paired-end reads were performed through the default parameters of SeqPrep.^1^ Then the HISAT2^2^ software was used to compare the clean reads with the Ningxiang pig reference genome (accession number PPJNA531381) in a orientation mode. The mapped readings of each sample were assembled by StringTie^3^ in a reference-based method. After comparing with HISAT2, it is assembled by stringtie software. Compare with the known transcripts in the known genome annotation information (Gffcompare software) to obtain new transcripts, and select the classcode as “x,” “i,” “j,” “u,” “o” transcript. Transcripts with length ≥200 bp, Exon number ≥2, and ORF length ≤300 bp were selected as candidate lncRNAs for preliminary screening. Whether a transcript has coding potential is a key condition for judging whether a transcript is an lncRNA. The most widely used coding potential analysis method for lncRNA screening mainly includes four methods: CPC analysis, CNCI analysis, CPAT analysis, and Pfam protein domain analysis. The default screening conditions for these four methods are: CPC score <0.5; CNCI score <0; CPAT score <0.5; Pfam database has no annotations. The overlap analysis of lncRNA predicted by these four methods was carried out. Finally, the result of the new prediction of lncRNA takes the intersection of these softwares as the basis for subsequent analysis. Those with coding capabilities that are not predicted to be lncRNAs will be classified as new mRNAs.

### Data Construction and Processing of miRNA Libraries

One microgram total RNA was extracted from the tissue as the starting template. The small RNA sequencing library was prepared using TruSeq Small RNA Sample Prep Kits (Illumina, San Diego, CA, United States). The random primer was reversed into first cDNA (TruSeQ^TM^ Small RNA Sample Prep Kit). Library purification to remove adaptor dimer, junk, low complexity, common RNA families (rRNA, tRNA, snRNA, snoRNA), and repeat sequences (6% Novex TBE PAGE Gel, 1.0 mm, 10 well). TBS380 (Picogreen) quantitative, according to the proportion of data mixed on the computer. The constructed library was sequenced using Illumina Hiseq 2000/2500, and the length of sequenced reads was 1 × 50 bp with single end. Compare clean reads with miRNA precursor and mature body sequences in the miRBase database of selected wild boar species. Align the sRNA sequences that cannot be compared on Rfam and miRBase to the reference genome, intercept the surrounding sequences and use miRDeep2 software to predict the secondary structure. According to the prediction results, use Dicer restriction site information, energy value, and other characteristics to filter to identify new miRNAs.

### The Prediction of Target Genes of miRNAs

In order to predict the genes targeted by miRNAs, we used two computational target prediction algorithms, miRanda 3.3a (score cutoff ≥160 and energy cutoff 20), and RNAHybrida (energy cutoff = 20) to jointly identify the binding sites of miRNAs. Finally, the data predicted by the two algorithms are combined and the overlapping target genes are calculated.

### Analysis of Differentially Expressed Genes and Differentially Expressed miRNAs

Salmon (Patro et al., 2017) was used to perform expression level for unigenes by calculating Transcripts Per Million (TPM) (Mortazavi et al., 2008). DEMs and DEGs were identified by the DEseq2 with *p*-adjust < 0.05 and had fold-change ≥2 with N90 vs. N30 d, N210 vs. N30 d and N210 vs. N90 d. DEPs were identified by the *t*-test with *P*-value < 0.05 and had fold-change ≥1.2 or ≤0.83 with N90 vs. N30 d, N210 vs. N30 d, and N210 vs. N90 d. Gene function was annotated based on the following databases: NCBI non-redundant protein sequences (Nr,ftp^4^), Protein Family (Pfam^5^), String,^6^ and (KEGG^7^).

### Time-Series Analysis

Time-series analysis was performed by STEM (Ernst and Bar-Joseph, 2006). The STEM analysis studies the dynamic behavior of gene expression and measures a series of processes that have a strong correlation with time points. The significantly enriched model profiles have a *P*-value of less than 0.05.

### Prediction of Target Genes of miRNAs, and Network Analysis

To define all the possible negative relationships between miRNA and mRNA expression. RNAhybrid and miRanda were used to predict the miRNA-gene pairs. The correlation between mRNAs and miRNAs was evaluated using the Pearson correlation coefficient (PCC) from matched mRNA and miRNA expression profile data. The interaction network was built and visually displayed using Cytoscape software. Protein identification was employed against the above RNA-seq results for database searching, the proteins added miRNA–mRNA pairs directly to the miRNA–mRNA–protein (Morris et al., 2015).

### Mass Spectrometry Analysis

All samples were analyzed using Q Exactive HF-X mass spectrometer (Thermo, United States) and UltiMate 3000 RSLC nanosystem (Thermo, United States). The peptide was injected onto a self-made C18 column (75 μm × 25mm, Thermo, United States) in buffer A (2% acetonitrile and 0.1% Formic acid) and separated with a linear gradient of buffer B (80% acetonitrile and 0.1% Formic acid) at a flow rate of 300 nl/min. Through nanoelectrospray ionization and Q-Exactive HF-X quadrupole orbit rap mass spectrometer interface, the integrated column heater is set at 50°C, and the electrospray voltage is 1.8 kV. The tandem mass spectra of the first 20 ions were scanned with a full range of 350–1300 m/z, the isolation width was set to 1.6 Da, the resolution was 70K, and the MS/MS scan resolution was 17.5K. In all cases, one microscan was recorded using dynamic exclusion of 18 s.

### Sequence Database Selection and Searching

Proteome Discoverer TM Software 2.2 software was used for database search identification and quantitative analysis against the Ningxiang pig database (accession number PPJNA531381). Since the original mass spectrometer off-machine data was a raw file, the raw file is submitted to the Proteome Discoverer server, and the Ningxiang pig genome database is selected for database search. The parameters for protein searching were the default parameter. Trypsin digestion with up to two missed cleavages. The carbamoylmethylation of cysteine is used as a fixed modification, and the oxidation of methionine and the acetylation of the protein N-terminus were used as variable modifications. Based on the *q*-value, a 1% false discovery rate (FDR) was used to verify the peptide spectral matching.

### Predicted miRNA–mRNA–Protein Validation by qRT-PCR and PRM

To validate the identified RNAs in Ningxiang pigs, total RNA was extracted using an Animal Total RNA Kit (Tiangen, China). Total RNA was extracted from the three stages (30, 90, and 210 days) livers used in the RNA-seq and reversed to cDNA with a Primescript RT Master Kit (Thermo Scientific, United States) with random primers in accordance with the manufacturer’s instructions. The PCR amplicon template sequence described above was then used to design the PCR primers using Primer 5 software. Primer sequences used in the qRT-PCR are listed in Supplementary File 10. All reactions were performed in triplicate for each sample. The relative expression level of the miRNAs and mRNAs was calculated *via* the comparative CT method (2^–△△^^CT^). U6/GAPDH was used as an internal control.

Mass spectrometric data was collected using the Triple TOF5600 and liquid mass spectrometry system (SCIEX). For PRM data collection, the optimized protein peptide list was added to the inclusion list. The peptides in the list were selected and separated one by one by mass spectrometry, and the secondary ion spectrum was collected. First, a part of all the peptide samples obtained after trypsin digestion were mixed for mass spectrometric DDA detection. The resulting MS–MS data were processed by Protein pilot^TM^ V4.5 search engine, then were processed with Skyline software and imported into the corresponding spectra library (MacLean et al., 2010). Screening the target protein peptide and establishing the method. The PRM detection method was designed by adding the target peptide m/z to the inclusion list to establish a mass spectrometry acquisition method. The mixed sample’s PRM data was collected by the PRM method to adjust and optimize the PRM data acquisition method and form the final PRM method for sample data acquisition. The optimized PRM mass spectrometry acquisition method was used to collect data for each sample, and PRM spectrum files was obtained. Through the extraction and analysis of these PRM map files, the quantitative information of protein can be obtained.

### Statistical Analysis

An unpaired two-tailed Student’s *t*-test was used to test changes in candidate differential miRNAs and mRNAs levels between any two time points. ^∗^*P* < 0.05, ^∗∗^*P* < 0.01. Data were expressed as mean ± SEM.

## Data Availability Statement

The datasets presented in this study can be found in online repositories. The names of the repository/repositories and accession number(s) can be found in the article/Supplementary Material.

## Ethics Statement

The animal study was reviewed and approved by all animal experiments in this study were approved by the “Institutional Animal Care and Use Committee of Hunan Agricultural University” (Changsha, China).

## Author Contributions

BL performed the data analysis and wrote the manuscript. QZ and JiH provided the site of experiment and some of test conditions support. YX and YG conducted part of animal experiments. YX completed the animal feeding experiment. YD, JY, KX, JuH, and HM designed this study and revised the manuscript. All authors contributed to the article and approved the submitted version.

## Conflict of Interest

QZ is employed by Dalong Livestock Technology Co., Ltd. The remaining authors declare that the research was conducted in the absence of any commercial or financial relationships that could be construed as a potential conflict of interest.

## Publisher’s Note

All claims expressed in this article are solely those of the authors and do not necessarily represent those of their affiliated organizations, or those of the publisher, the editors and the reviewers. Any product that may be evaluated in this article, or claim that may be made by its manufacturer, is not guaranteed or endorsed by the publisher.

## Footnotes

1
https://github.com/OpenGene/fastp

2
https://ccb.jhu.edu/software/hisat2/index.shtml

3
https://ccb.jhu.edu/software/stringtie/index.shtml

4
http://ftp.ncbi.nlm.nih.gov/blast/db/

5
http://pfam.xfam.org/

6
https://string-db.org

7
http://www.genome.jp/kegg/

## Supplementary Material

Supplementary Figure 1 All second-order mass spectrum matching figures of target protein candidate peptides.

Supplementary Figure 2 Skyline analysis of target peptide PRM results.

Supplementary Figure 3 Validation of the expression of DEGs/DEMs by qRT-PCR. Data from qRT-PCR are shown as column and *Y*-axis on the left, while the data from RNA-seq are shown as line and *Y*-axis on the right. Data are represented as mean ± SEM, *n* = 3 per group.

Supplementary Figure 4 Validation of the expression of DEPs by parallel reaction monitoring (PRM).

Supplementary File 1 List of expressed genes and miRNA during Ningxiang pig liver development. Gene ID, TPM value, gene name, GO/KO ID, position, and description of all acquired genes.

Supplementary File 2 List of all miRNA sequences identified during Ningxiang pig liver development.

Supplementary File 3 Quality control analysis of the miRNAome.

Supplementary File 4 List of expressed proteins during Ningxiang pig liver development. Protein ID, abundance value, description of all acquired proteins.

Supplementary File 5 Kyoto Encyclopedia of Genes and Genomes pathways for upregulated and downregulated DEPs from N90 vs. N30 d and N210 vs. N90 d. Pathways marked gray background represent significant enrichment, while other terms represent non-significant enrichment.

Supplementary File 6 Kyoto Encyclopedia of Genes and Genomes pathways were identified by KEGG pathway analysis of hepatic overlapping differentially expressed (DE) mRNAs from three comparison groups.

Supplementary File 7 The relationship of miRNAs and mRNAs regulatory networks based on profile III–profile IV.

Supplementary File 8 List of protein interaction network node attribute.

Supplementary File 9 Data analysis of PRM quantitative in the target peptide section. The yellow bottom represents the internal control protein alpha-actinin-1.

Supplementary File 10 Primers of quantitative real-time RT-PCR.

## Abbreviations

ncRNA: non-coding RNA
miRNA: microRNA
N30 d: Ningxiang pig 30 days after birth
N90 d: Ningxiang pig 90 days after birth
N210 d: Ningxiang pig 210 days after birth
vs: versus
DEGs: differentially expressed mRNAs
DEMs: differentially expressed miRNAs
DEPs: differentially expressed proteins
FDR: false discovery rate
TPM: Transcripts Per Kilobase of exon model per Million mapped reads
KEGG: Kyoto Encyclopedia of Genes and Genomes
STEM: Short Time-series Expression Miner
RPM: reads per million mapped reads
RNA-seq: RNA sequencing
CIRI 2: CircRNA identifier 2
qRT-PCR: quantitative real-time RT-PCR
PRM: parallel reaction monitor.
