# Supplementary figures and images for: Integrated Analysis of Liver Transcriptome, miRNA, and Proteome of Chinese Indigenous Breed Ningxiang Pig in Three Developmental Stages Uncovers Significant miRNA–mRNA–Protein Networks in Lipid Metabolism
Source: Front Genet. 2021 Sep 16;12:709521. doi: 10.3389/fgene.2021.709521 (PMC8481880; doi:10.3389/fgene.2021.709521)

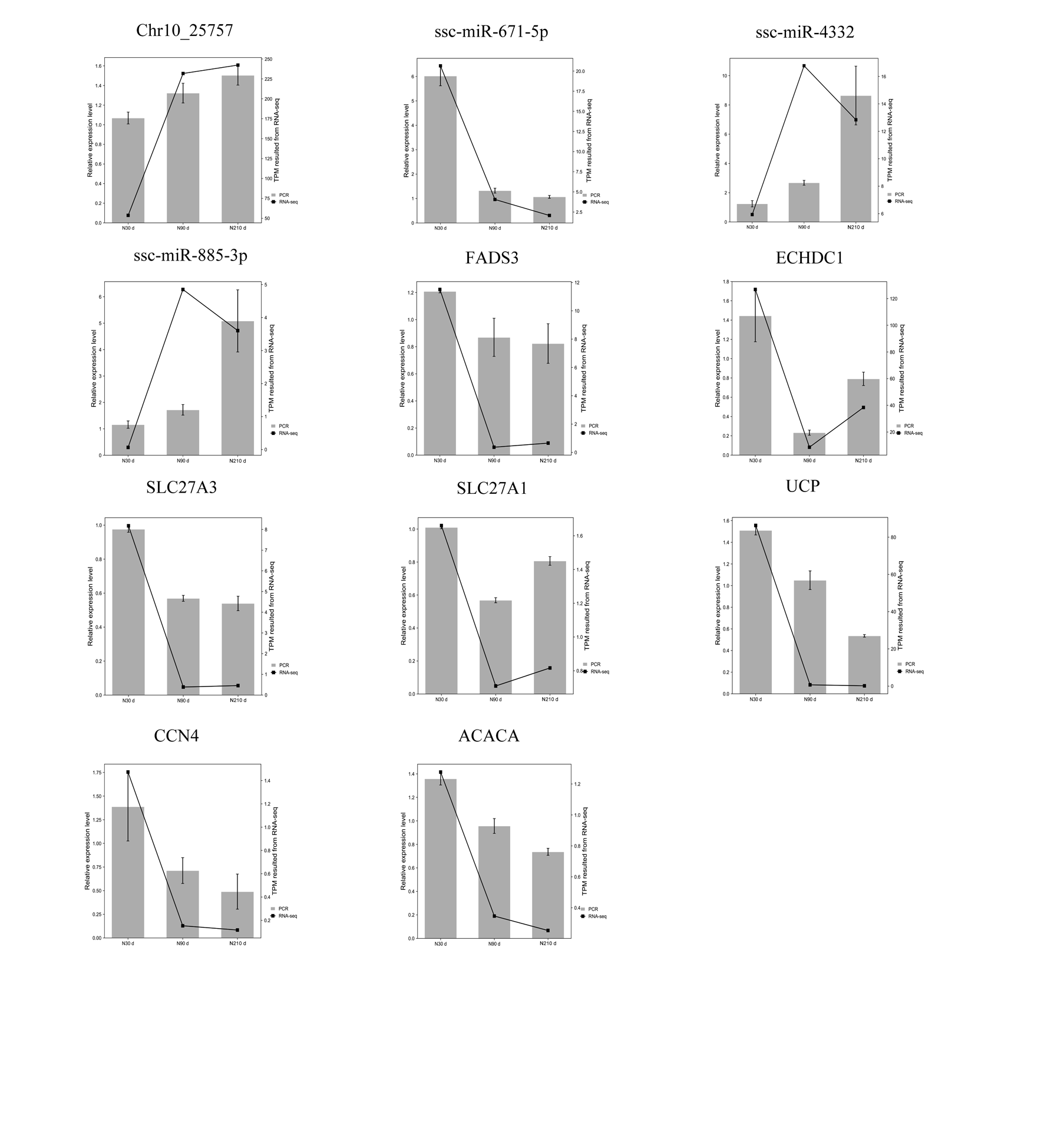

Supplement: Supplementary Figure 1 — All second-order mass spectrum matching figures of target protein candidate peptides. [file Image_1.TIF]

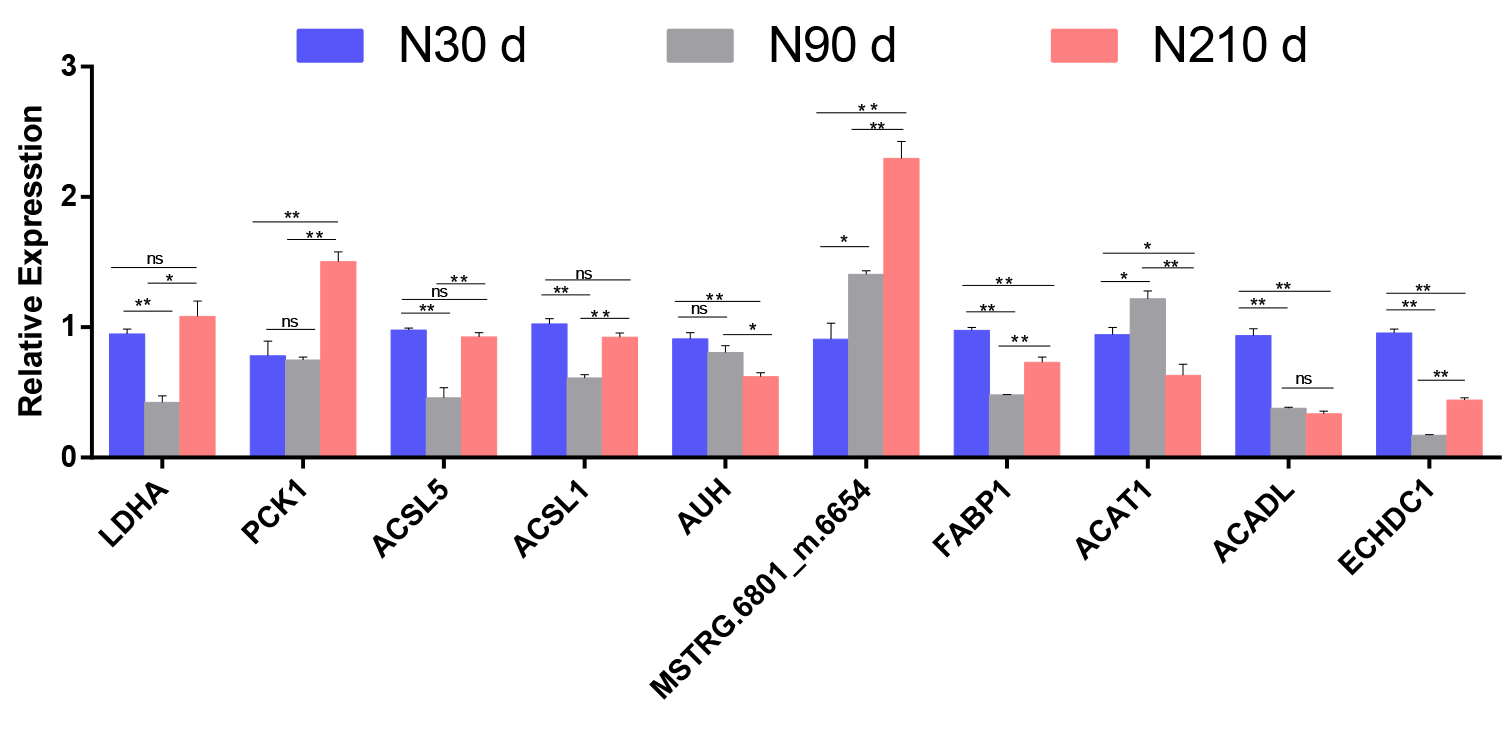

Supplement: Supplementary Figure 2 — Skyline analysis of target peptide PRM results. [file Image_2.TIF]
