# Supplementary material for: Integrated Analysis of Liver Transcriptome, miRNA, and Proteome of Chinese Indigenous Breed Ningxiang Pig in Three Developmental Stages Uncovers Significant miRNA–mRNA–Protein Networks in Lipid Metabolism
Source: Front Genet. 2021 Sep 16;12:709521. doi: 10.3389/fgene.2021.709521 (PMC8481880; doi:10.3389/fgene.2021.709521)
Supplement: Supplementary Figure 3 — Validation of the expression of DEGs/DEMs by qRT-PCR. Data from qRT-PCR are shown as column and Y-axis on the left, while the data from RNA-seq are shown as line and Y-axis on the right. Data are represented as mean ± SEM, n = 3 per group. [file Data_Sheet_1.PDF]

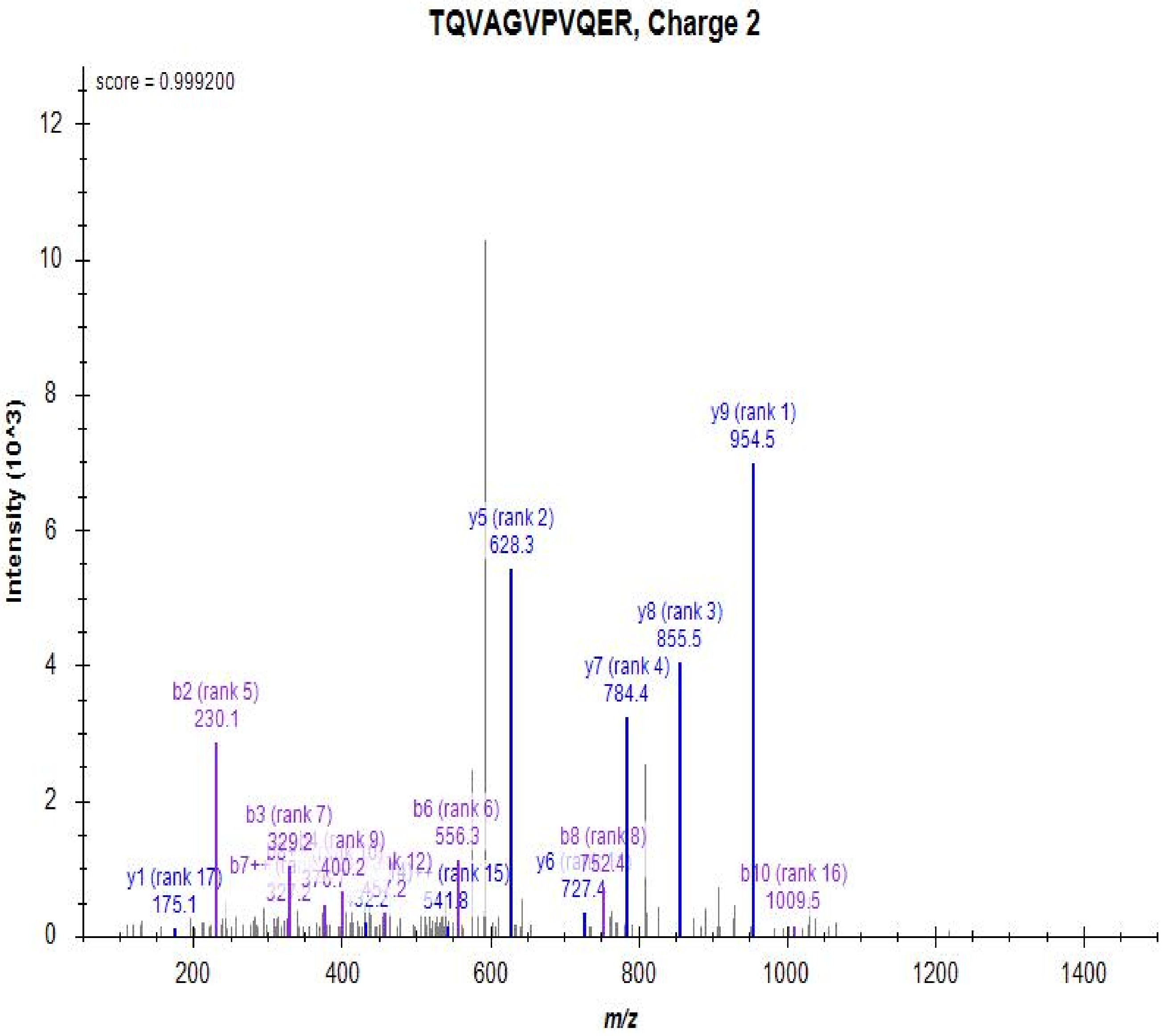

TQVAGVPVQER, Charge 2
score = 0.999200
Intensity (10^3)
m/z
y9 (rank 1)
954.5
y5 (rank 2)
628.3
y8 (rank 3)
855.5
y7 (rank 4)
784.4
b2 (rank 5)
230.1
b3 (rank 7)
329.2
b6 (rank 6)
556.3
b8 (rank 8)
752.4
400.2
y1 (rank 17)
175.1
b10 (rank 16)
1009.5
727.4
541.8

**EAILEDLLR, Charge 2**

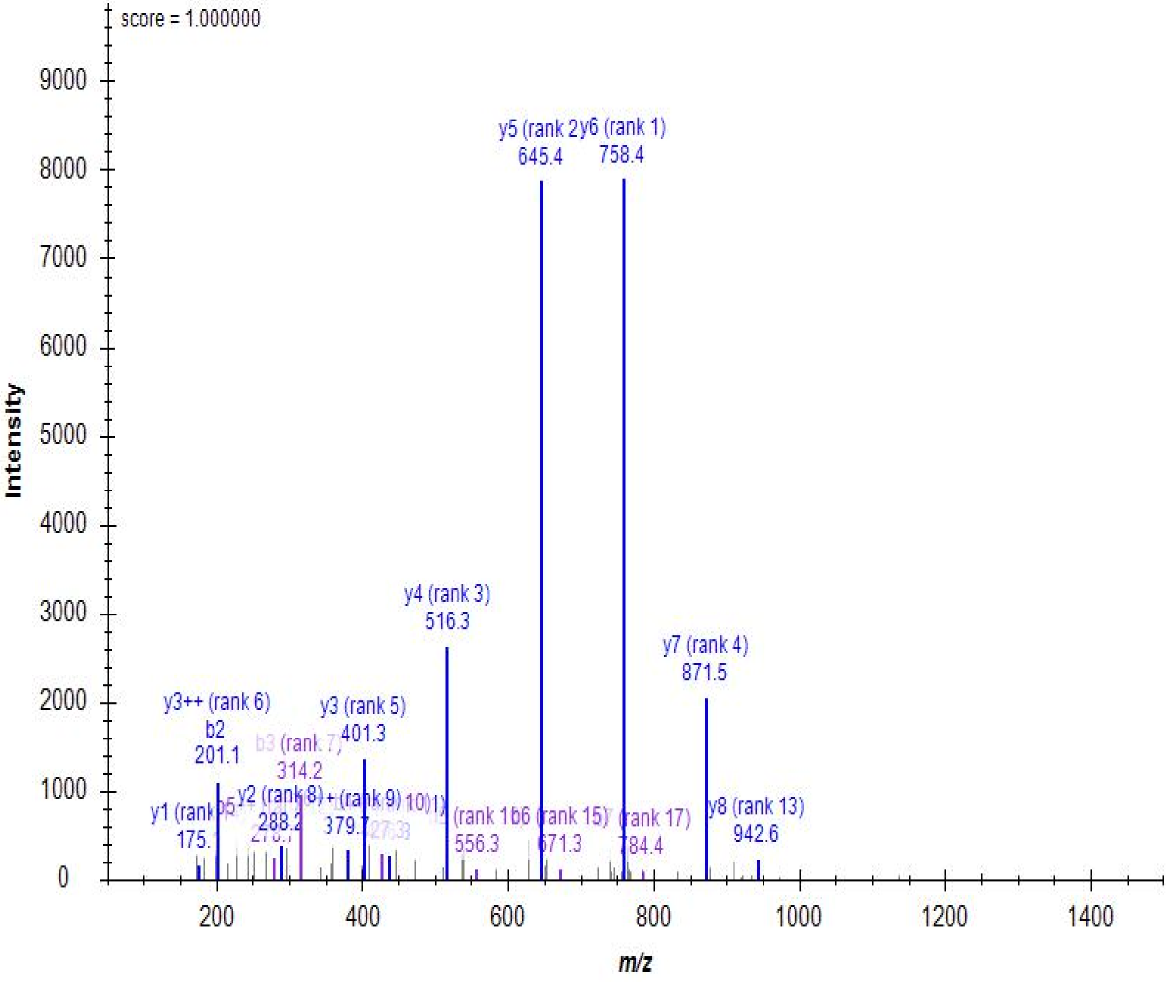

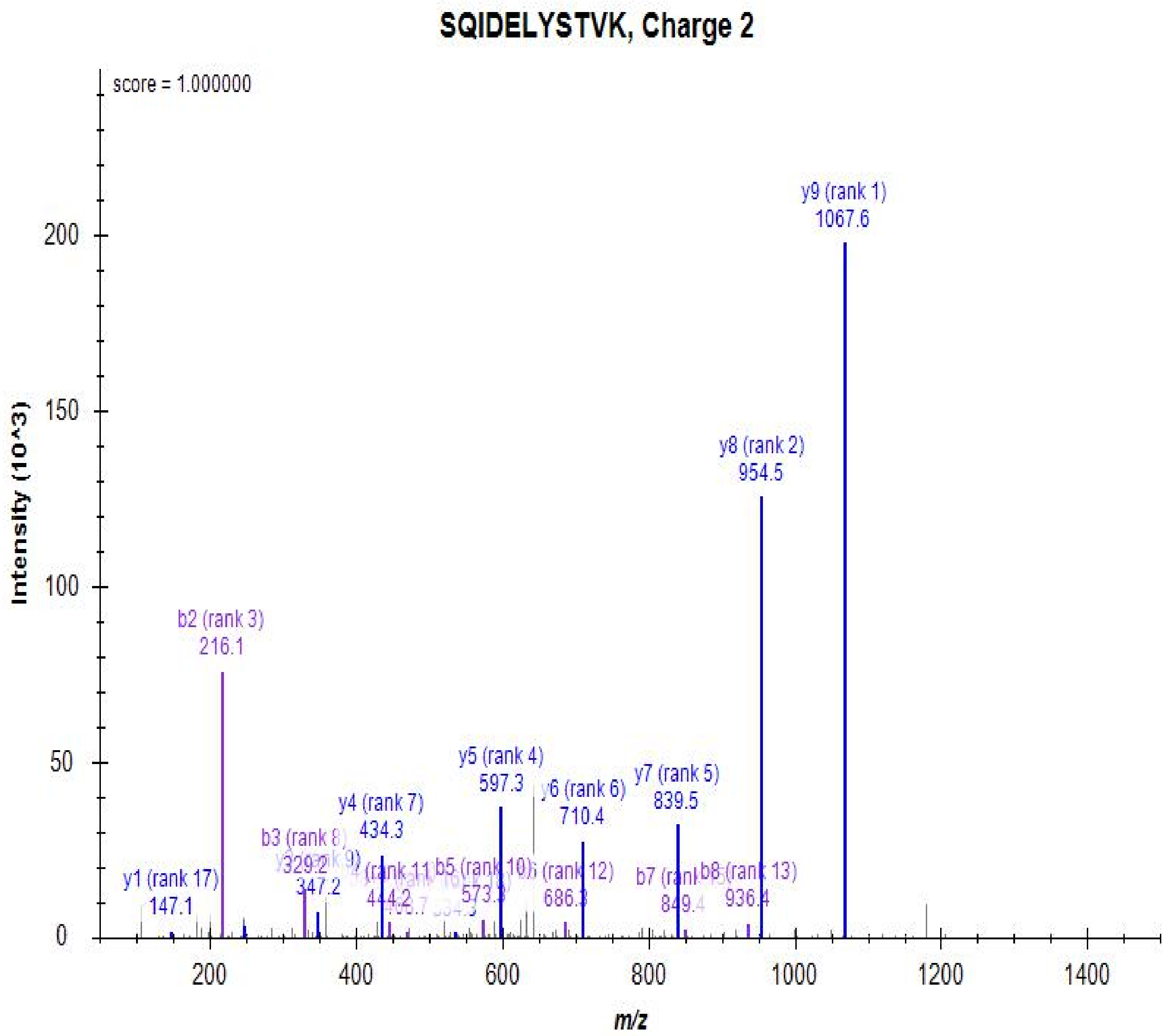
SQIDELYSTVK, Charge 2
score = 1.000000
Intensity (10^3)
m/z
y9 (rank 1)
1067.6
y8 (rank 2)
954.5
b2 (rank 3)
216.1
y5 (rank 4)
597.3
y7 (rank 5)
839.5
y6 (rank 6)
710.4
y4 (rank 7)
434.3
b3 (rank 8)
329.2
347.2
b5 (rank 10)
573.3
444.2
686.3
b8
936.4
849.4
y1 (rank 17)
147.1

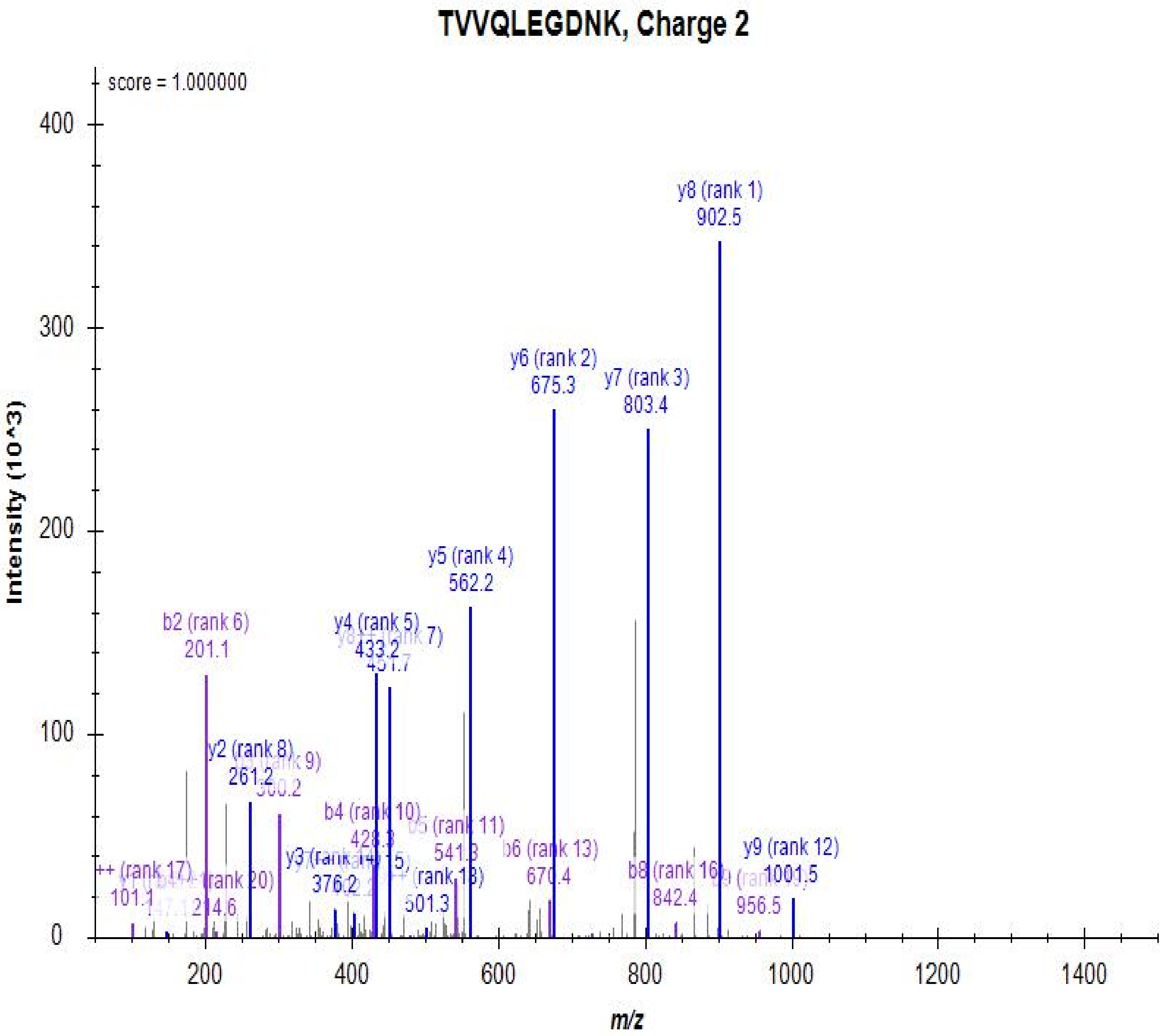

TVVQLEGDNK, Charge 2
score = 1.000000
Intensity (10^3)
m/z
y8 (rank 1)
902.5
y6 (rank 2)
675.3
y7 (rank 3)
803.4
y5 (rank 4)
562.2
y4 (rank 5)
433.2
b2 (rank 6)
201.1
y2 (rank 8)
261.2
b4 (rank 10)
b6 (rank 13)
670.4
y9 (rank 12)
1001.5
956.5
842.4
501.3
376.2
101.1

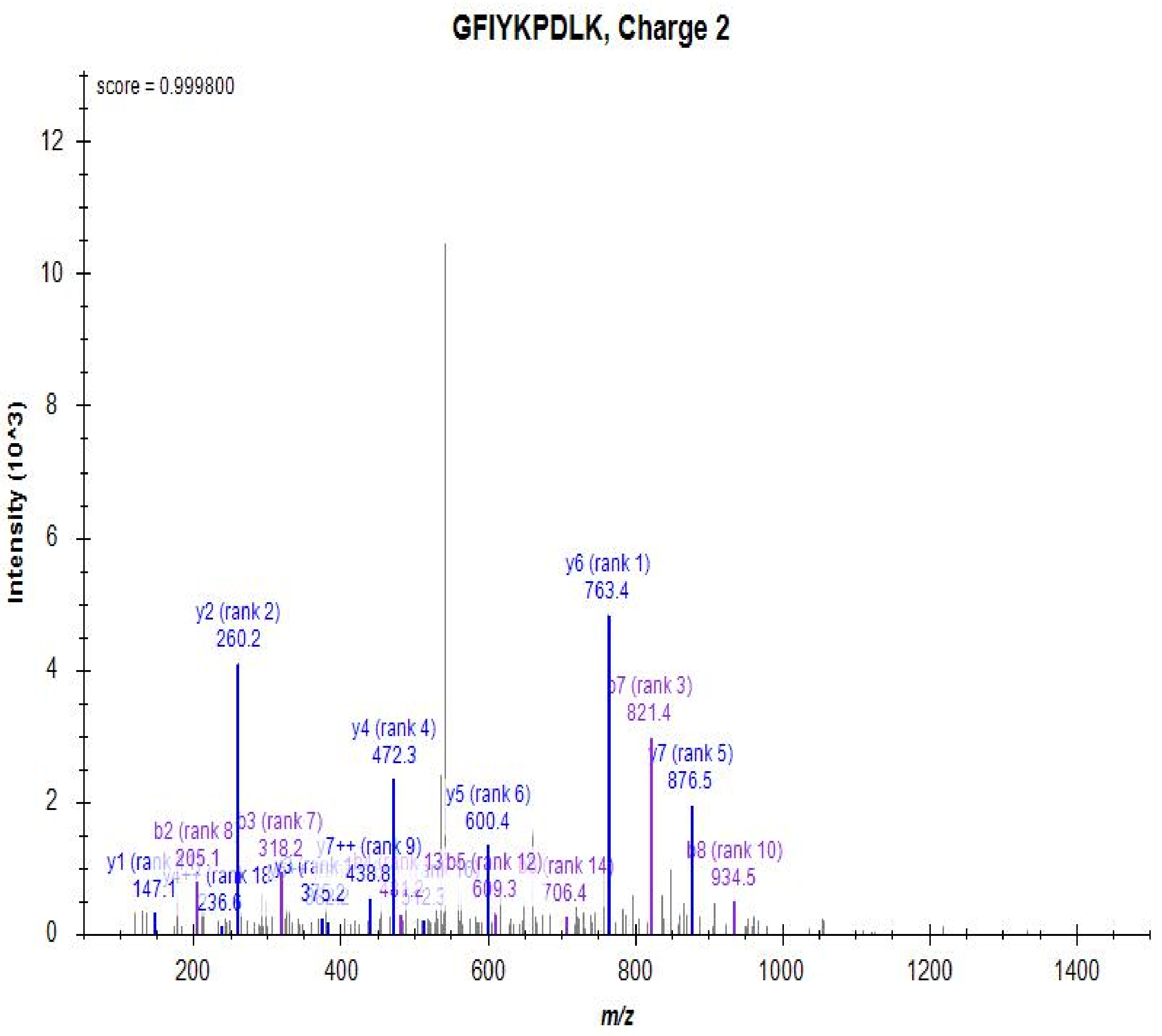
GFIYKPDLK, Charge 2
score = 0.999800
Intensity (10^3)
m/z
y6 (rank 1) 763.4
y2 (rank 2) 260.2
b7 (rank 3) 821.4
y4 (rank 4) 472.3
y7 (rank 5) 876.5
y5 (rank 6) 600.4
b3 (rank 7) 318.2
b2 (rank 8 205.1
y7++ (rank 9) 438.8
b8 (rank 10) 934.5
b5 (rank 12) 609.3
(rank 14) 706.4
y1 147.1
236.6
375.2

**AGLQEAGLAPVPMIIFAK, Charge 2**

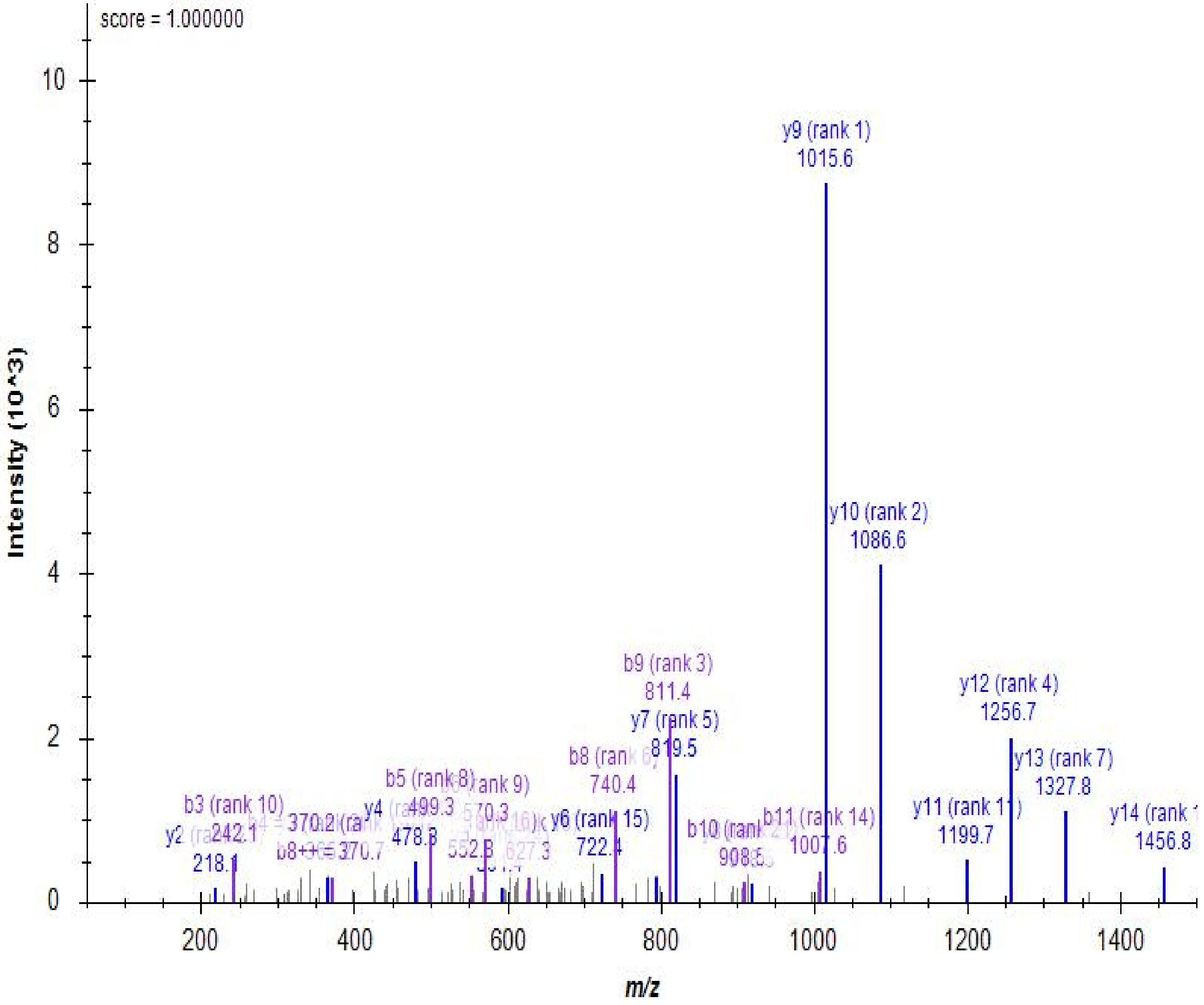

LFVYDPNNPPSSEVLR, Charge 2

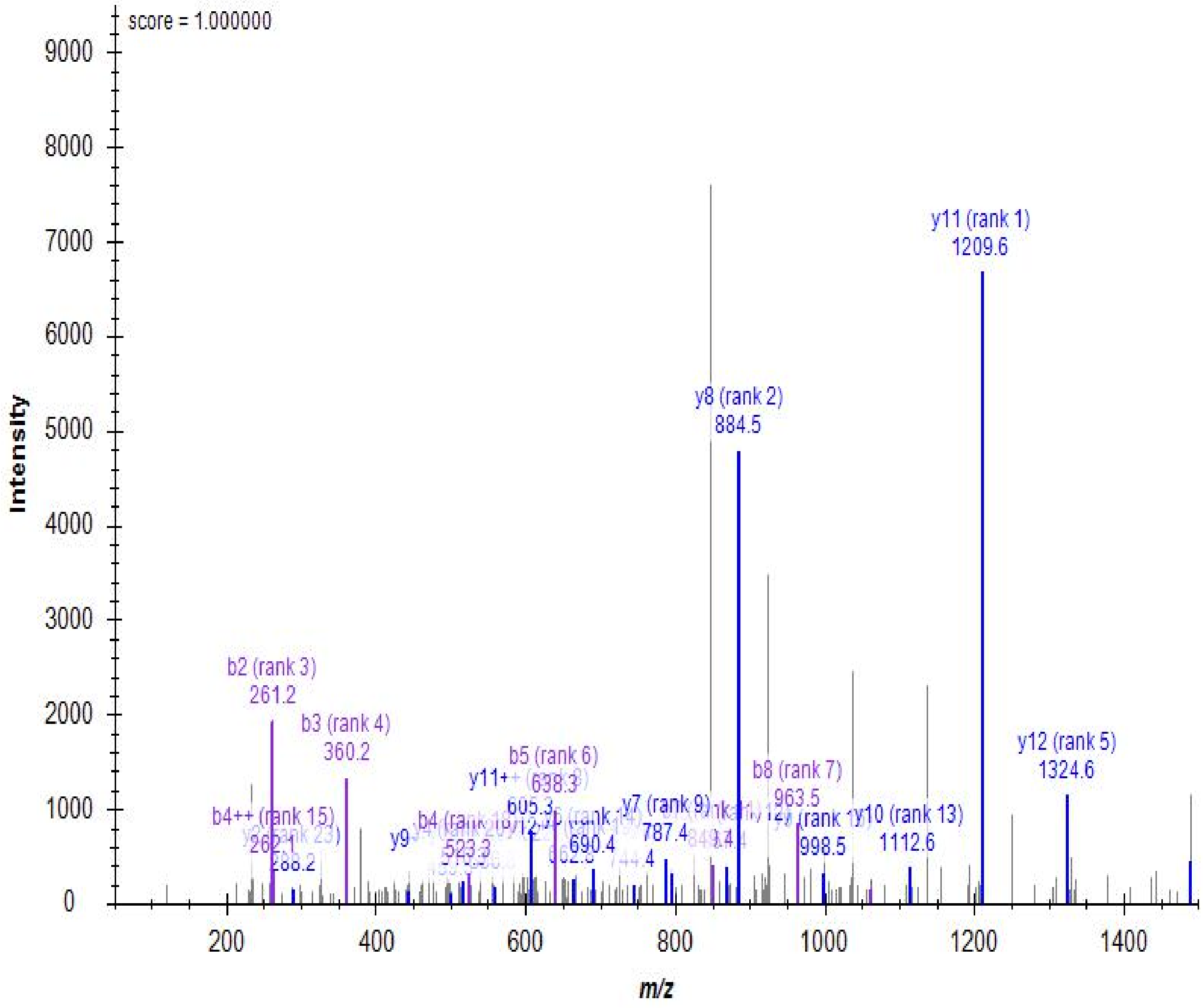

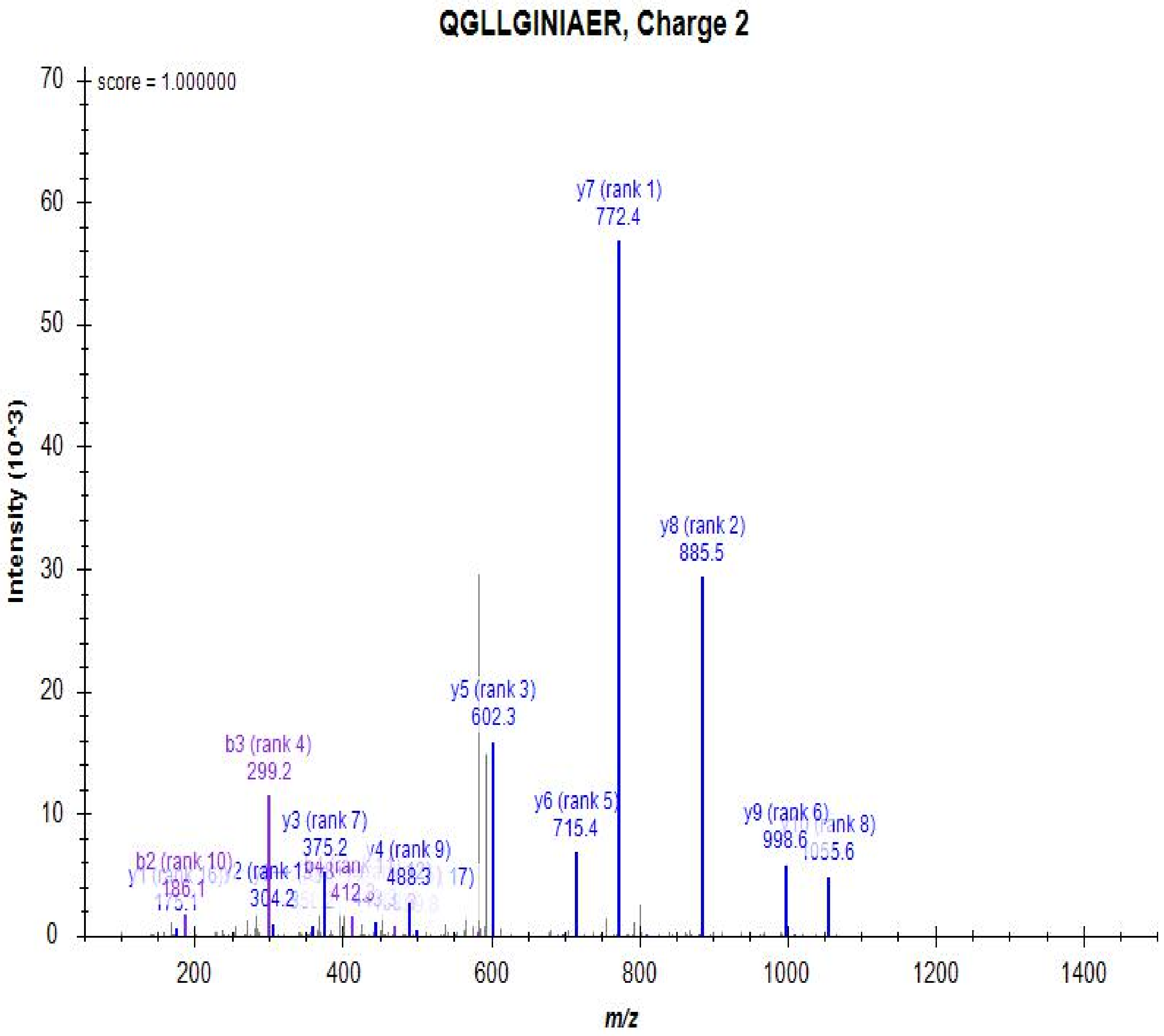
QGLLGINIAER, Charge 2
score = 1.000000
Intensity (10^3)
m/z
y7 (rank 1)
772.4
y8 (rank 2)
885.5
y5 (rank 3)
602.3
b3 (rank 4)
299.2
y6 (rank 5)
715.4
y9 (rank 6)
998.6
y3 (rank 7)
375.2
y4 (rank 9)
488.3
b2 (rank 10)
186.1
304.2
412.3
175.1
1055.6

LGSIAIQGAIEK, Charge 2

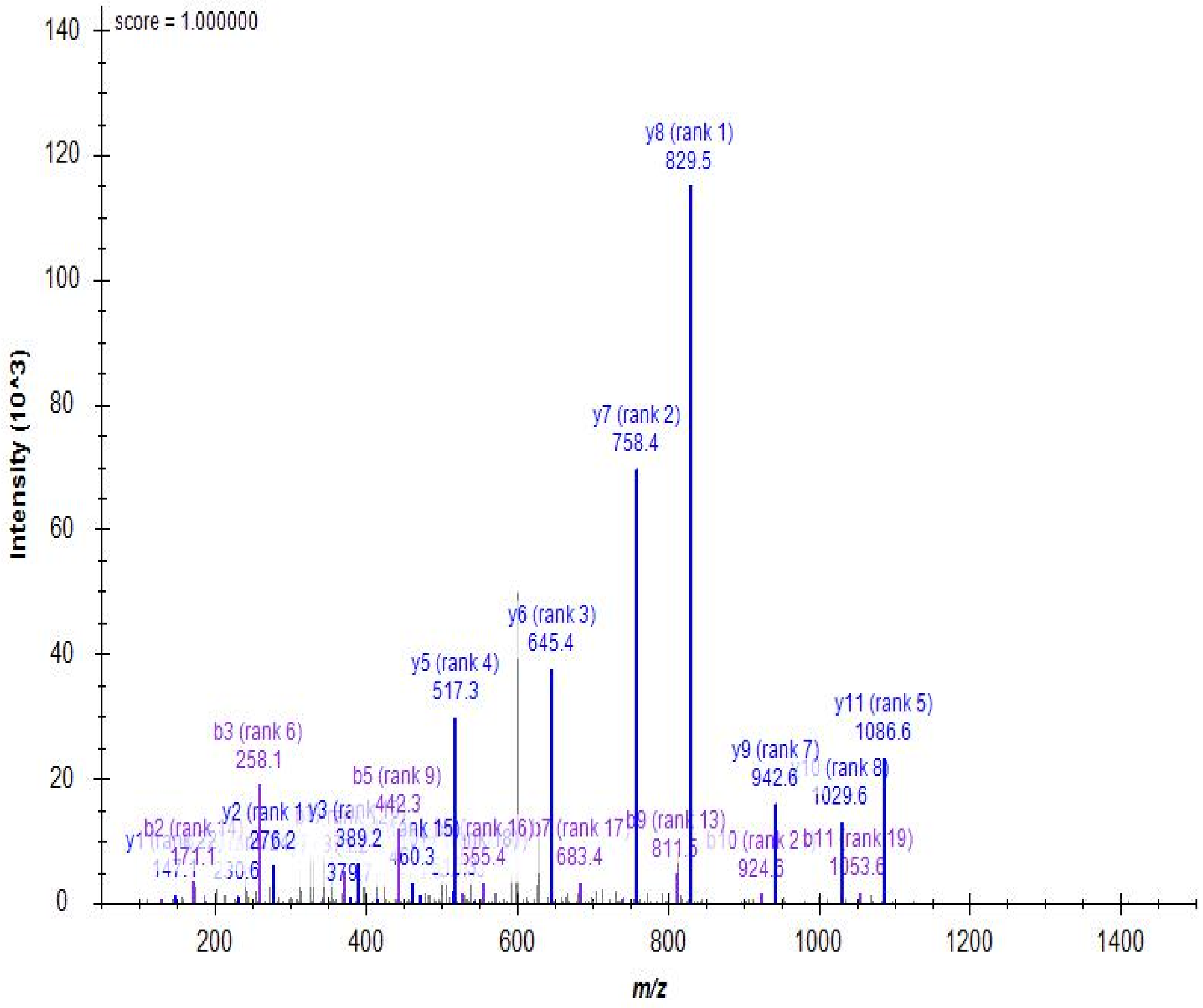

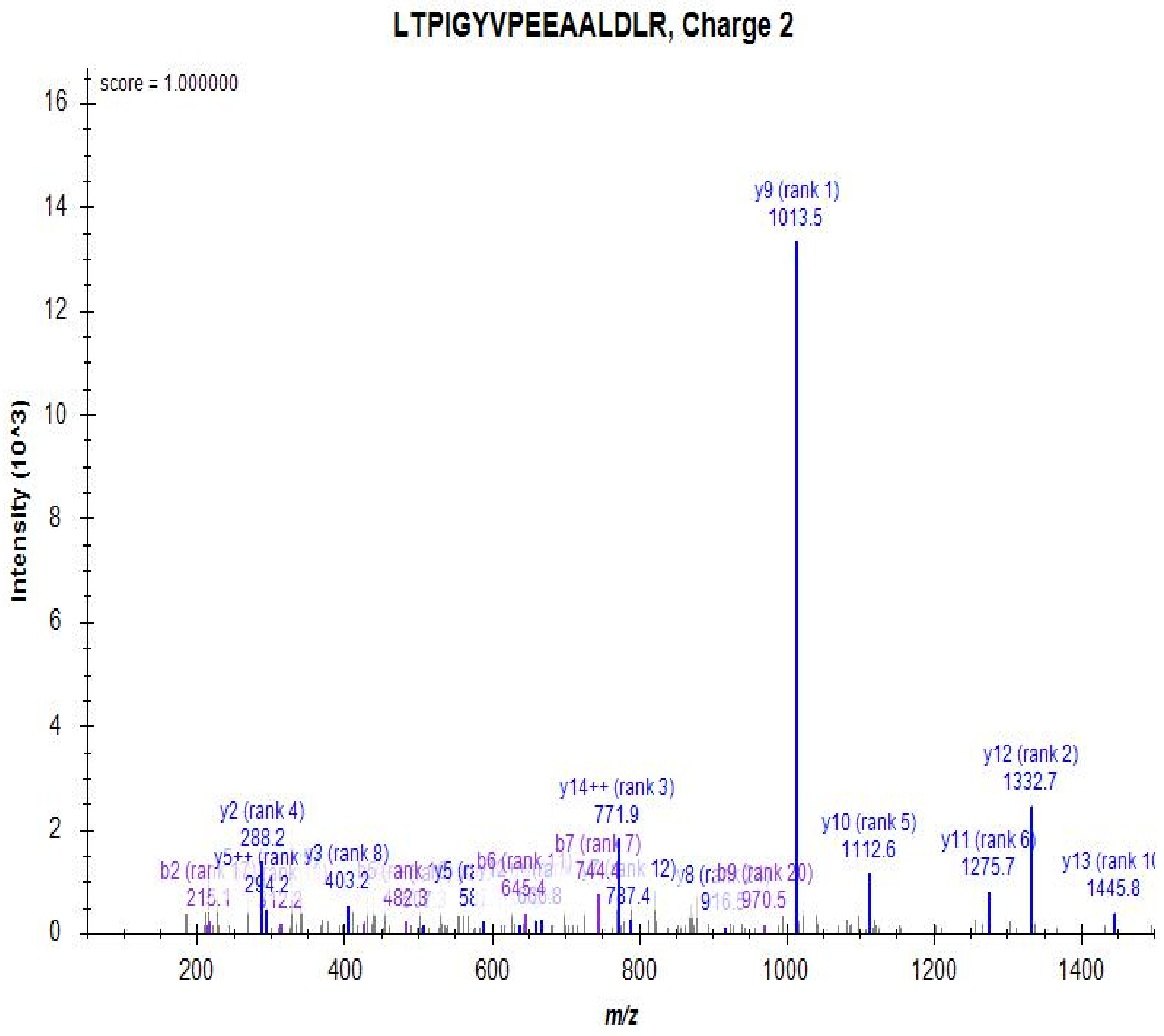

LTPIGYVPEEAALDLR, Charge 2
score = 1.000000
Intensity (10^3)
m/z
y9 (rank 1)
1013.5
y12 (rank 2)
1332.7
y14++ (rank 3)
771.9
y2 (rank 4)
288.2
y10 (rank 5)
1112.6
y11 (rank 6
1275.7
b7 (rank 7)
744.4
y3 (rank 8)
403.2
y13 (rank 1
1445.8
b9 (rank 20)
970.5
b6 (rank 11)
645.4
787.4
294.2
215.1
482.3
916.5

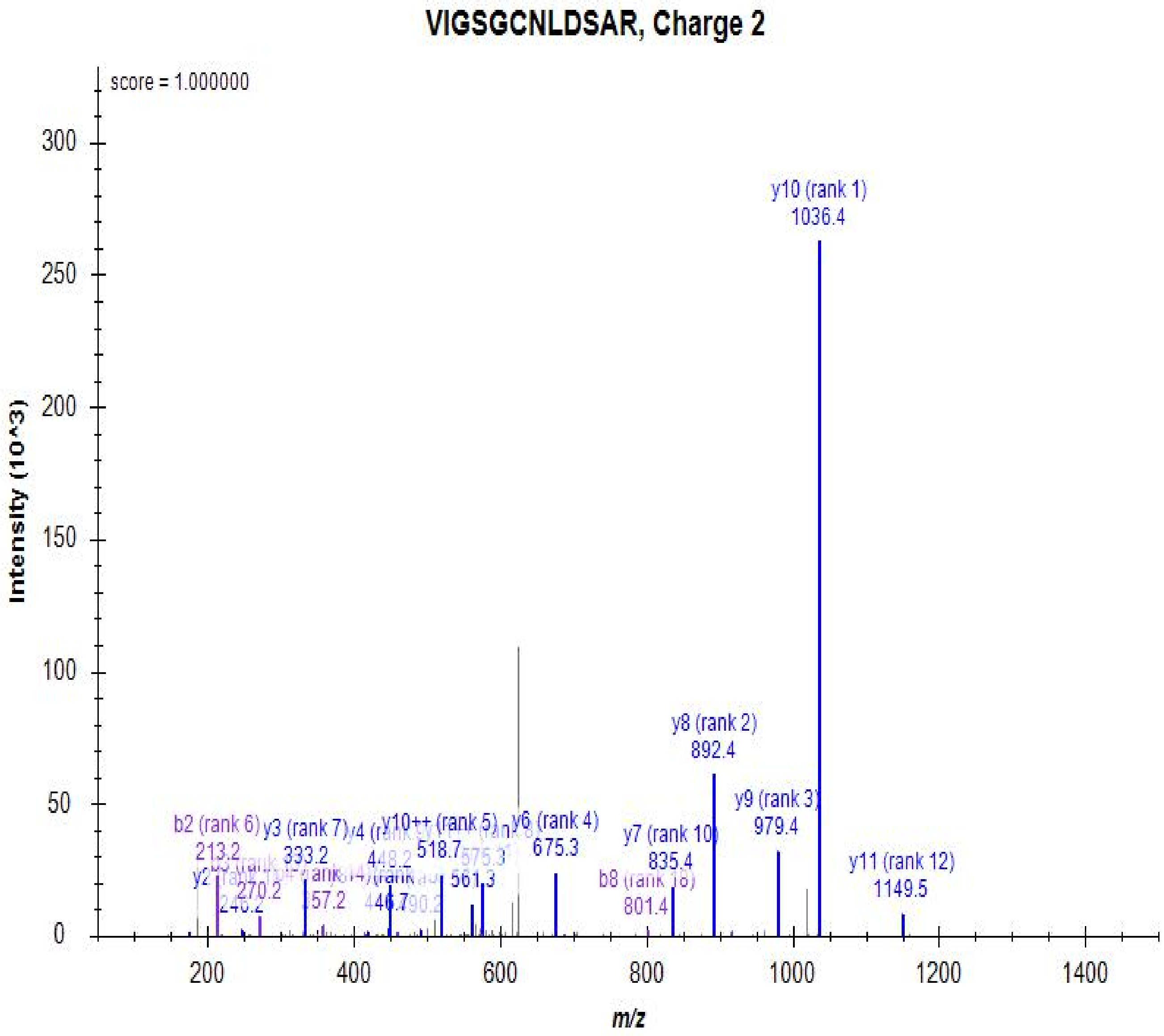
VIGSGCNLDSAR, Charge 2
score = 1.000000
Intensity (10^3)
m/z
y10 (rank 1)
1036.4
y8 (rank 2)
892.4
y9 (rank 3)
979.4
y6 (rank 4)
675.3
y10++ (rank 5)
518.7
b2 (rank 6)
213.2
y3 (rank 7)
333.2
y7 (rank 10)
835.4
y11 (rank 12)
1149.5
b8 (rank 18)
801.4
561.3
270.2
357.2
446.7
448.2
575.3
0
50
100
150
200
250
300
200
400
600
800
1000
1200
1400

# AYVEANQMLGDLIK, Charge 2

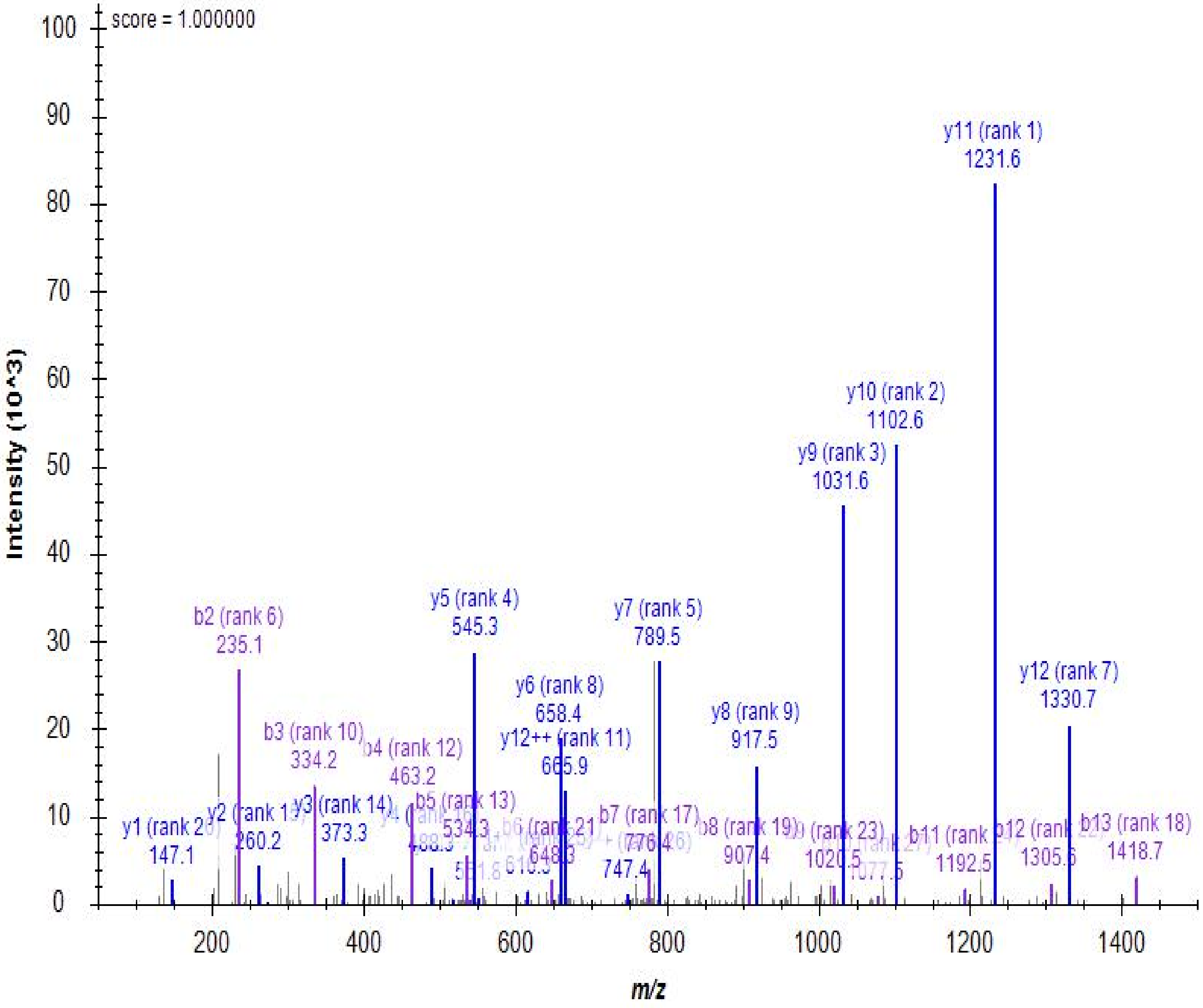

**DLLGTVWGGPANLEAVAR, Charge 2**

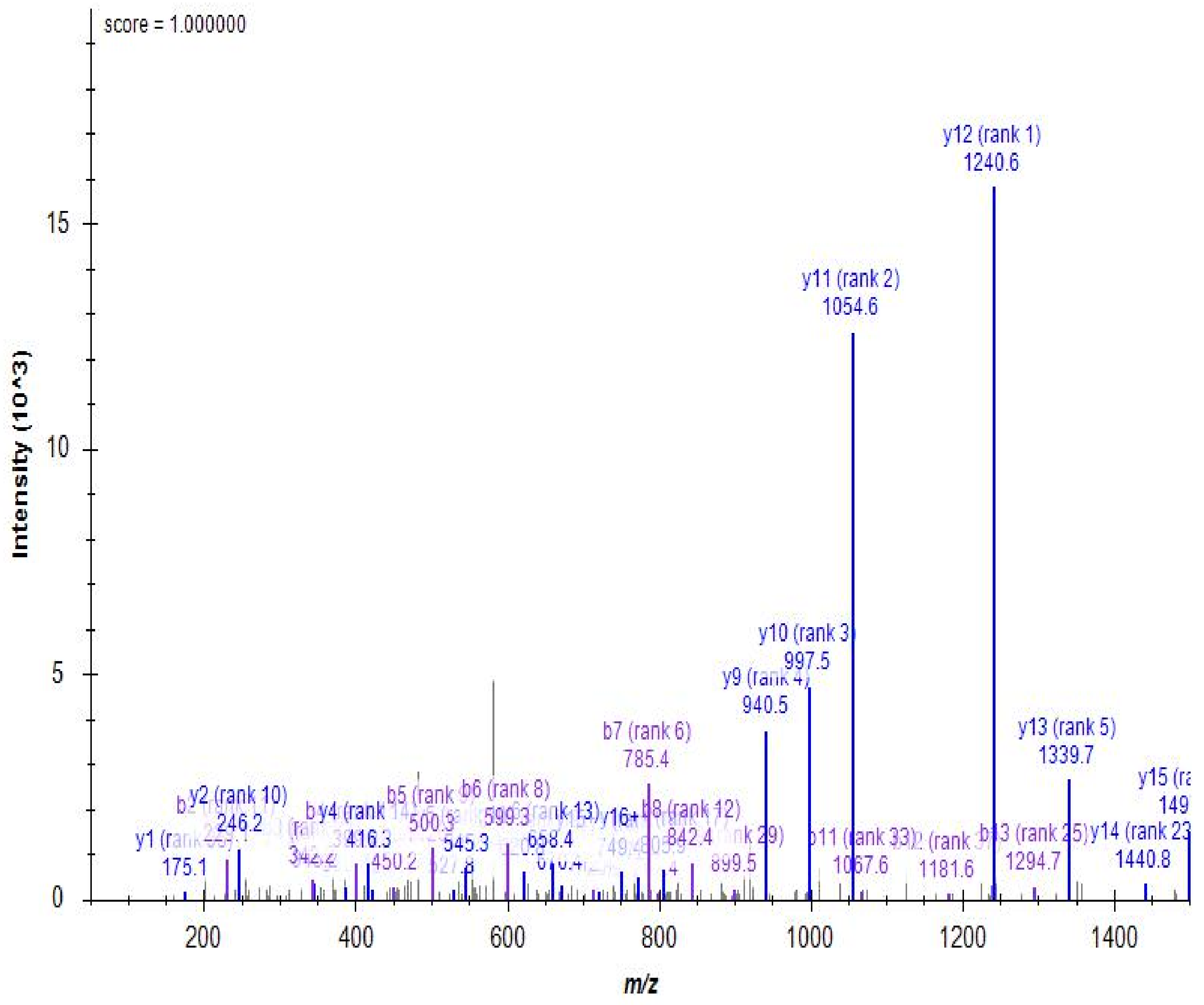

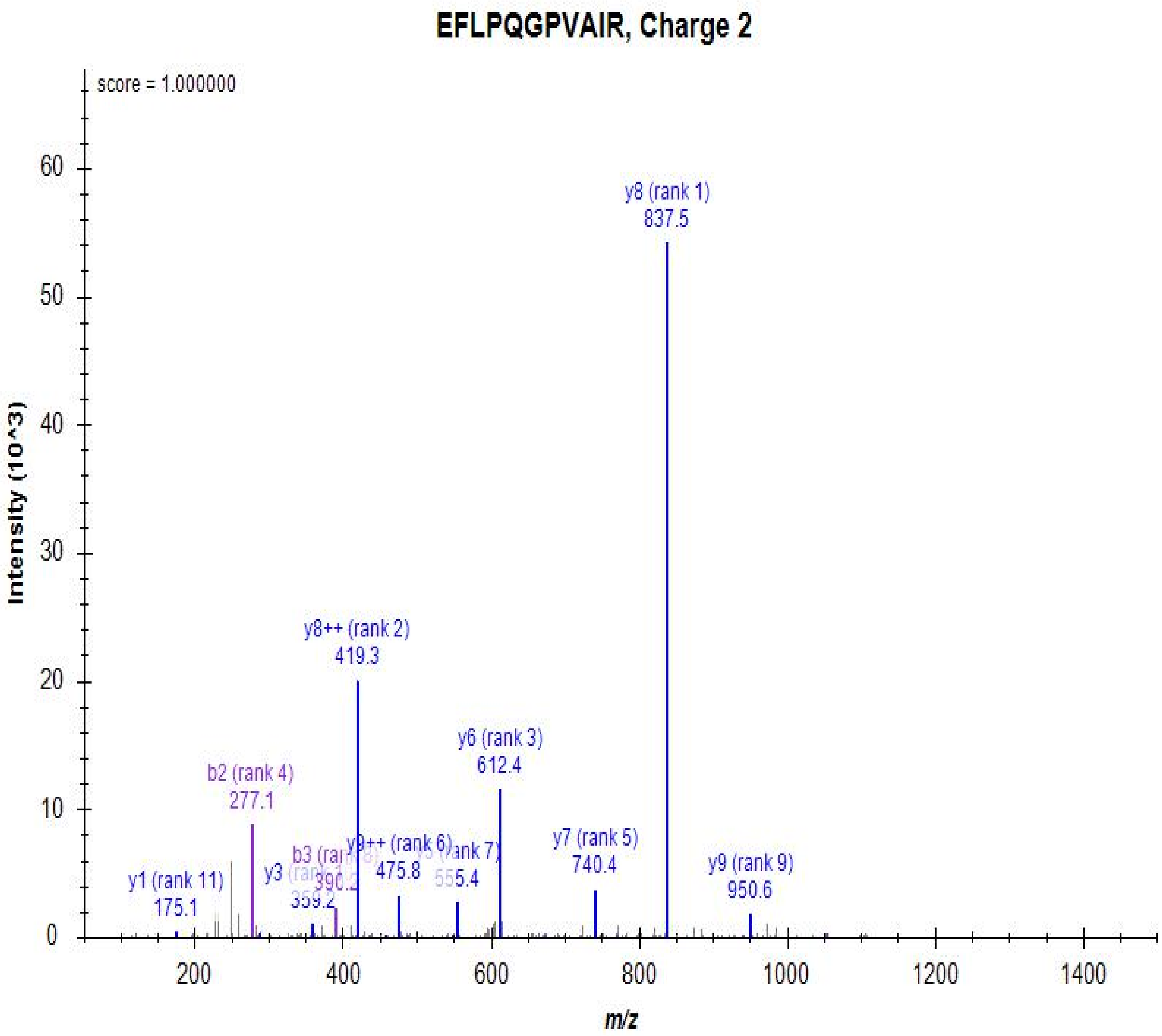

EFLPQGPVAIR, Charge 2
score = 1.000000
Intensity (10^3)
m/z
y8 (rank 1)
837.5
y8++ (rank 2)
419.3
y6 (rank 3)
612.4
b2 (rank 4)
277.1
y7 (rank 5)
740.4
y9++ (rank 6)
475.8
y9 (rank 9)
950.6
y1 (rank 11)
175.1
359.2
390.2
555.4

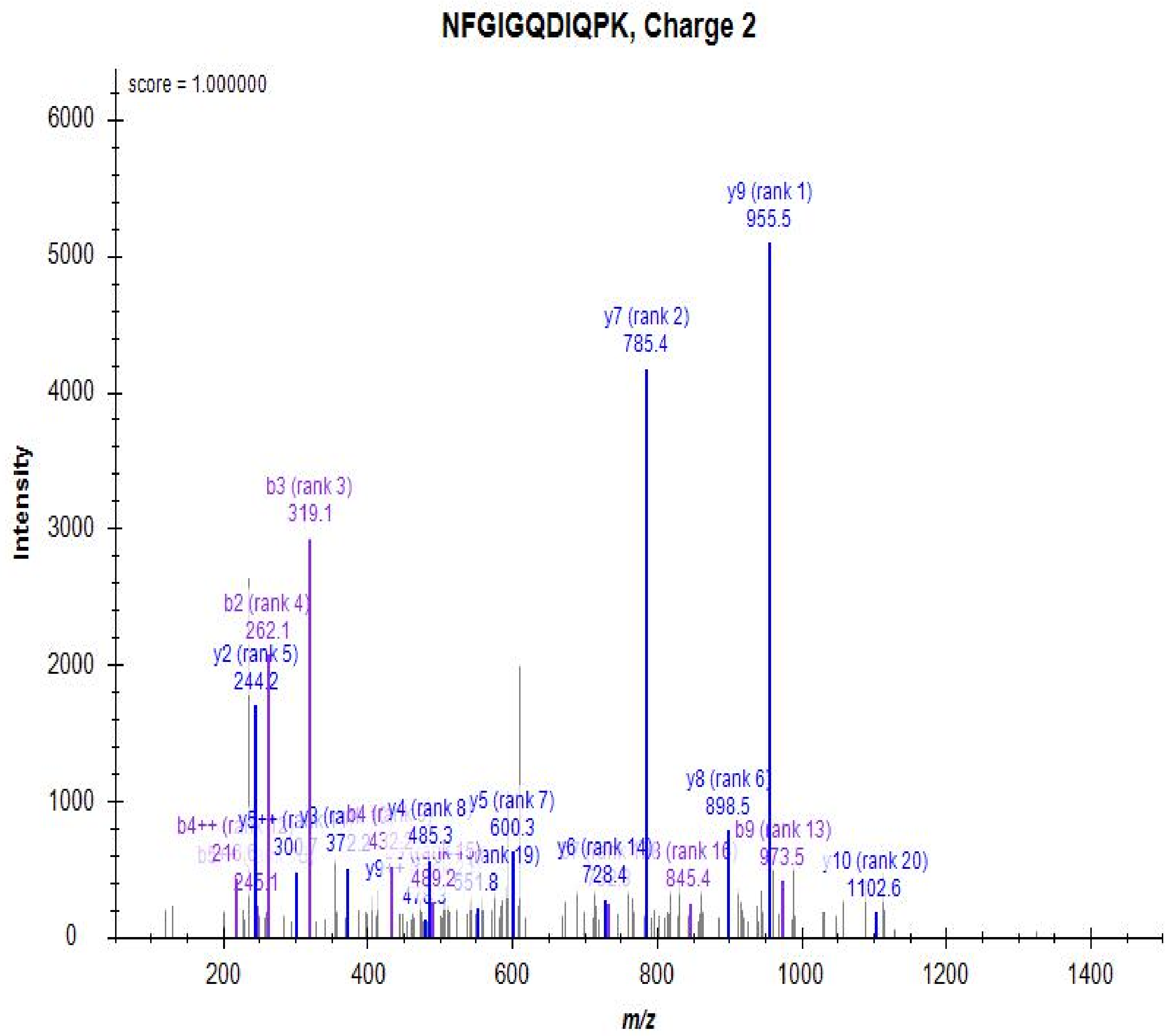
NFGIGQDIQPK, Charge 2
score = 1.000000
Intensity
m/z
y9 (rank 1)
955.5
y7 (rank 2)
785.4
b3 (rank 3)
319.1
b2 (rank 4)
262.1
y2 (rank 5)
244.2
y8 (rank 6)
898.5
y5 (rank 7)
600.3
y4 (rank 8
485.3
b9 (rank 13)
973.5
y6 (rank 14)
728.4
845.4
y10 (rank 20)
1102.6
372.2
432.2
489.2
551.8
216.6
245.1
300.7
