# Supplementary material for: Integrated Analysis of Liver Transcriptome, miRNA, and Proteome of Chinese Indigenous Breed Ningxiang Pig in Three Developmental Stages Uncovers Significant miRNA–mRNA–Protein Networks in Lipid Metabolism
Source: Front Genet. 2021 Sep 16;12:709521. doi: 10.3389/fgene.2021.709521 (PMC8481880; doi:10.3389/fgene.2021.709521)

Pig.20799.1(CHID1, K.TQVAGVPVQER.G [37, 47])

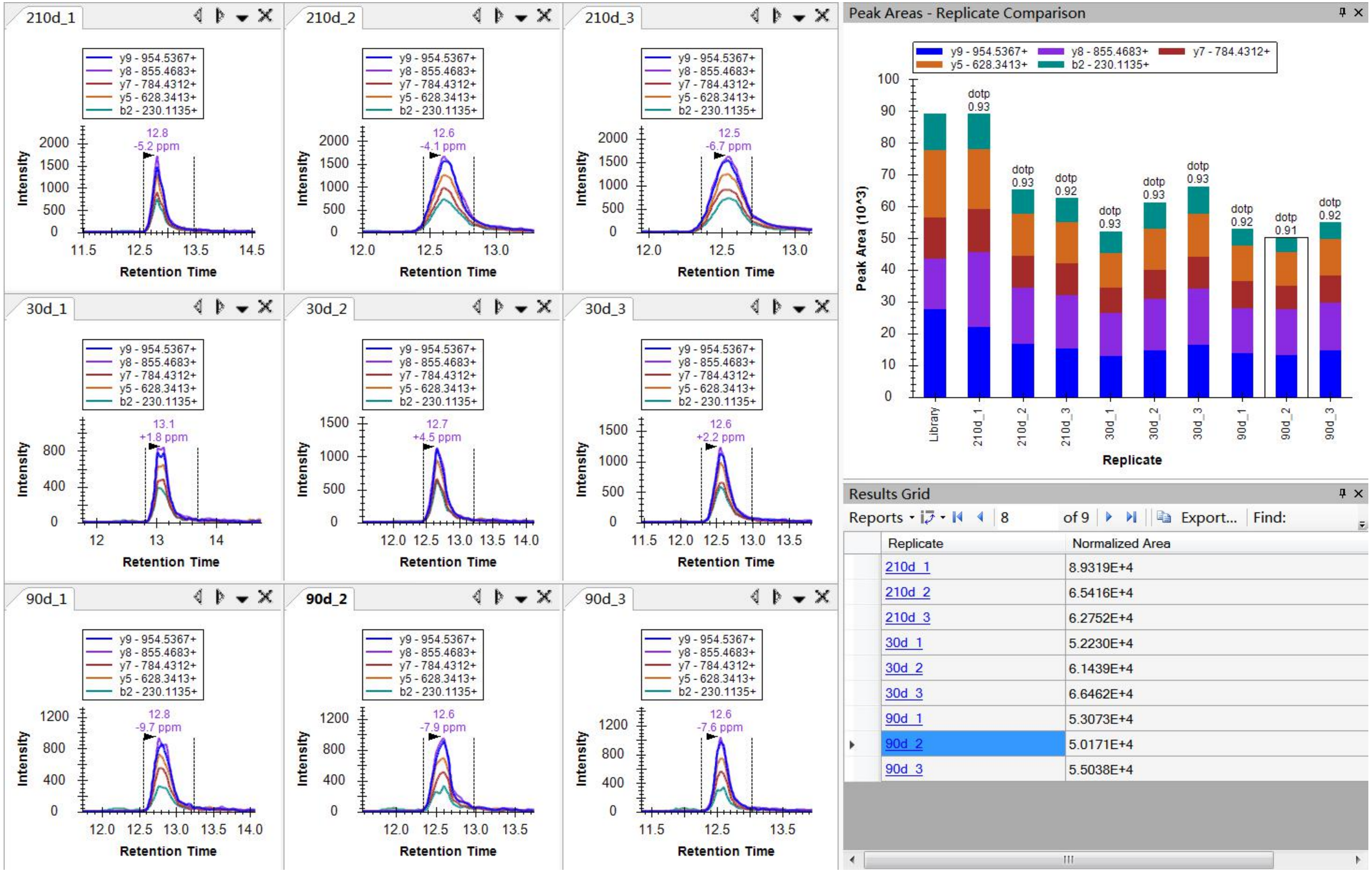

Pig.05131.1(ACSL5, K.EAILEDLLR.T [810, 818])

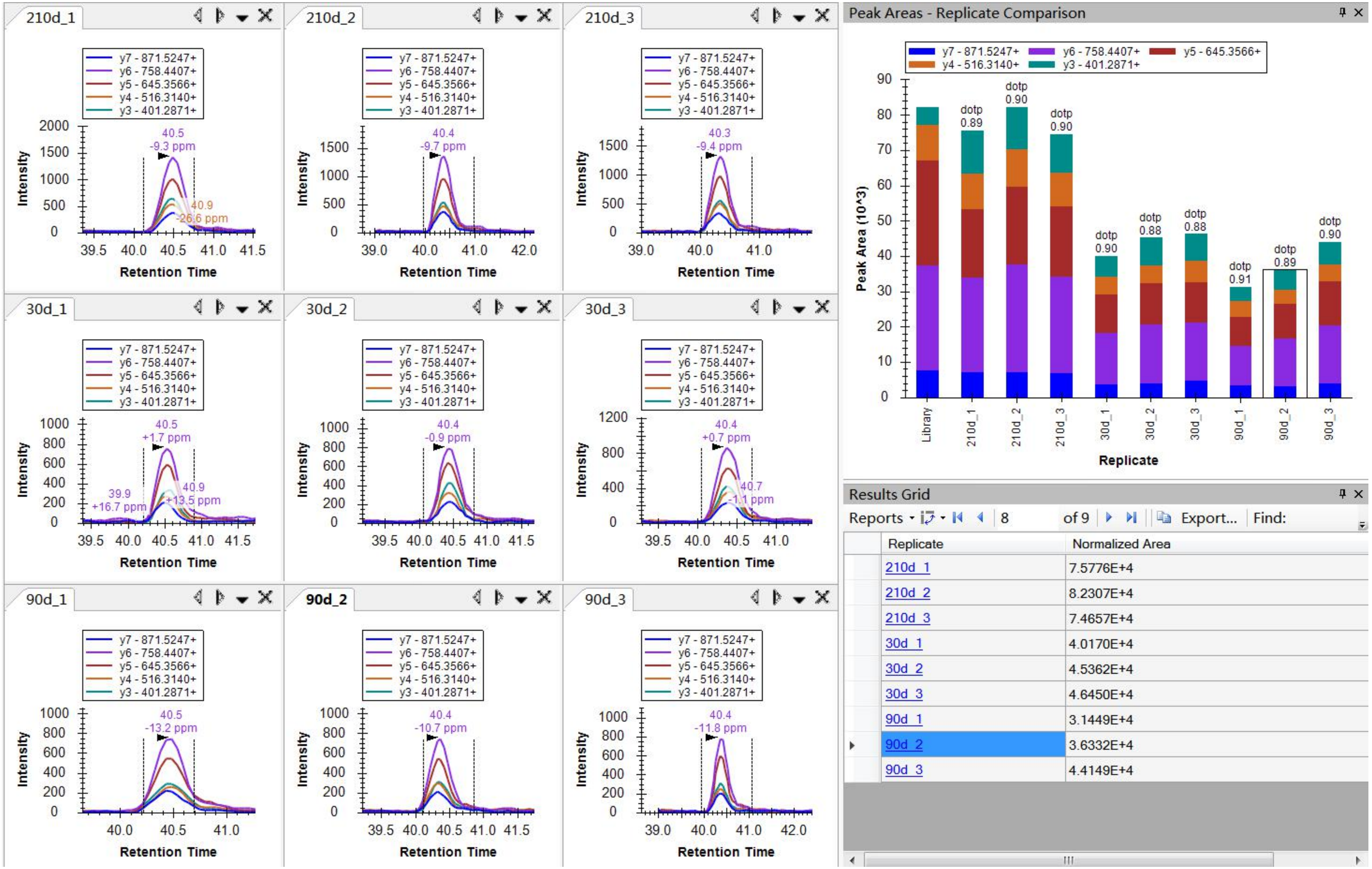

Pig.02709.1(ACSL1, R.SQIDELYSTVK.V [687, 697])

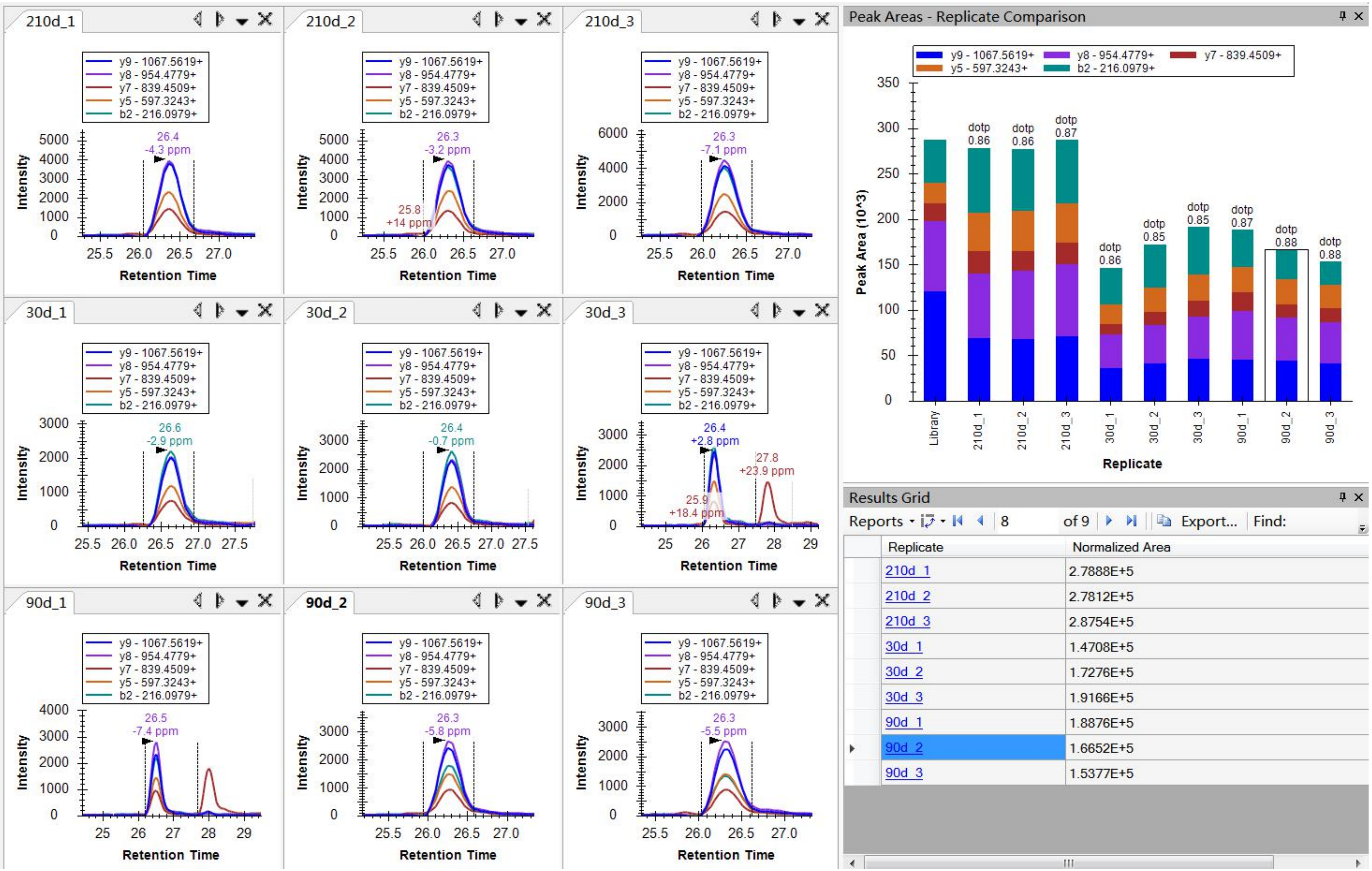

Pig.09406.1(FABP1, K.TVVQLEGDNK.L [80, 89])

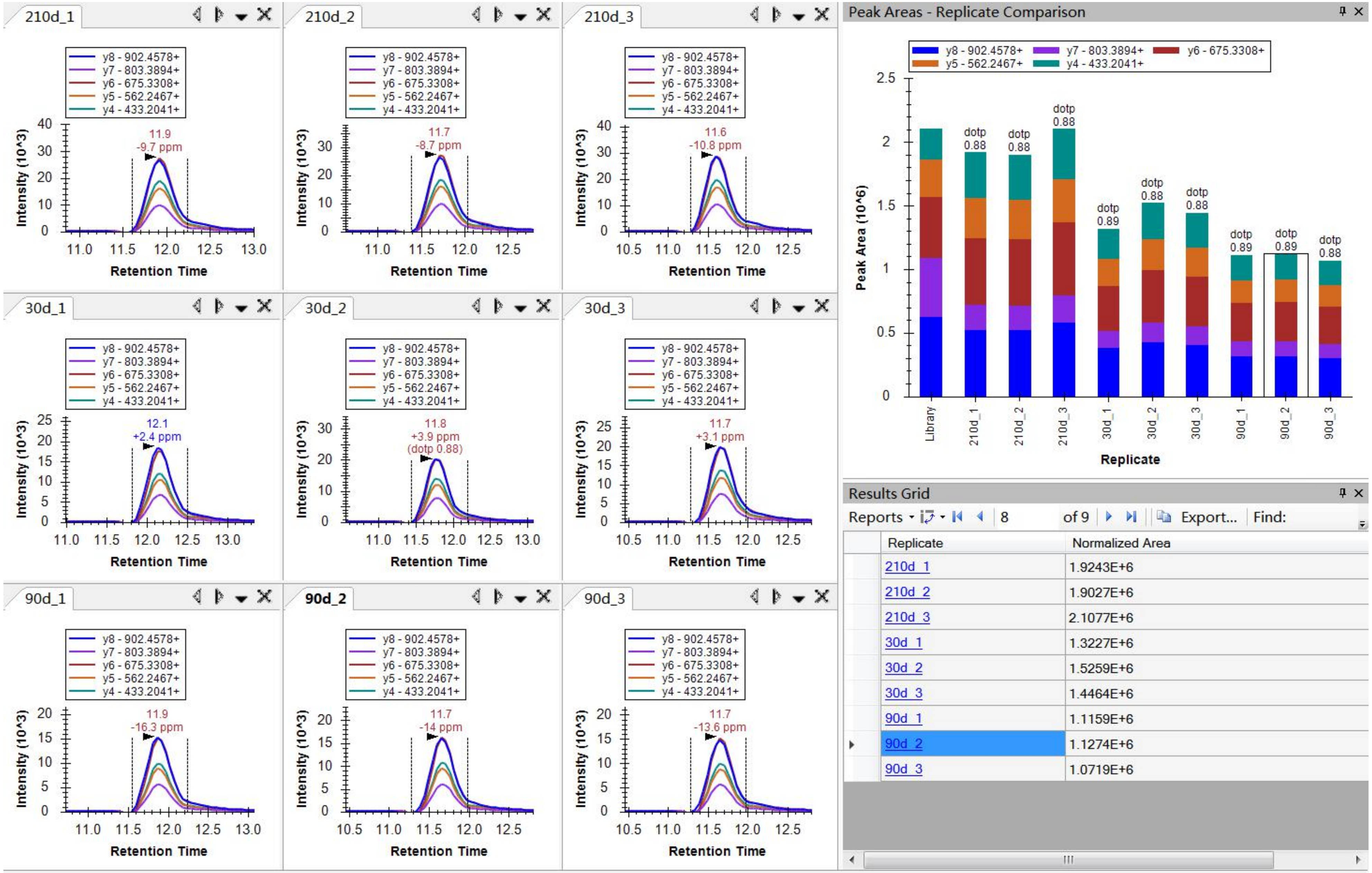

Replicate	Normalized Area
210d_1	1.9243E+6
210d_2	1.9027E+6
210d_3	2.1077E+6
30d_1	1.3227E+6
30d_2	1.5259E+6
30d_3	1.4464E+6
90d_1	1.1159E+6
90d_2	1.1274E+6
90d_3	1.0719E+6

Pig.17389.1(FBN1, K.GFIYKPDLK.T [794, 802])

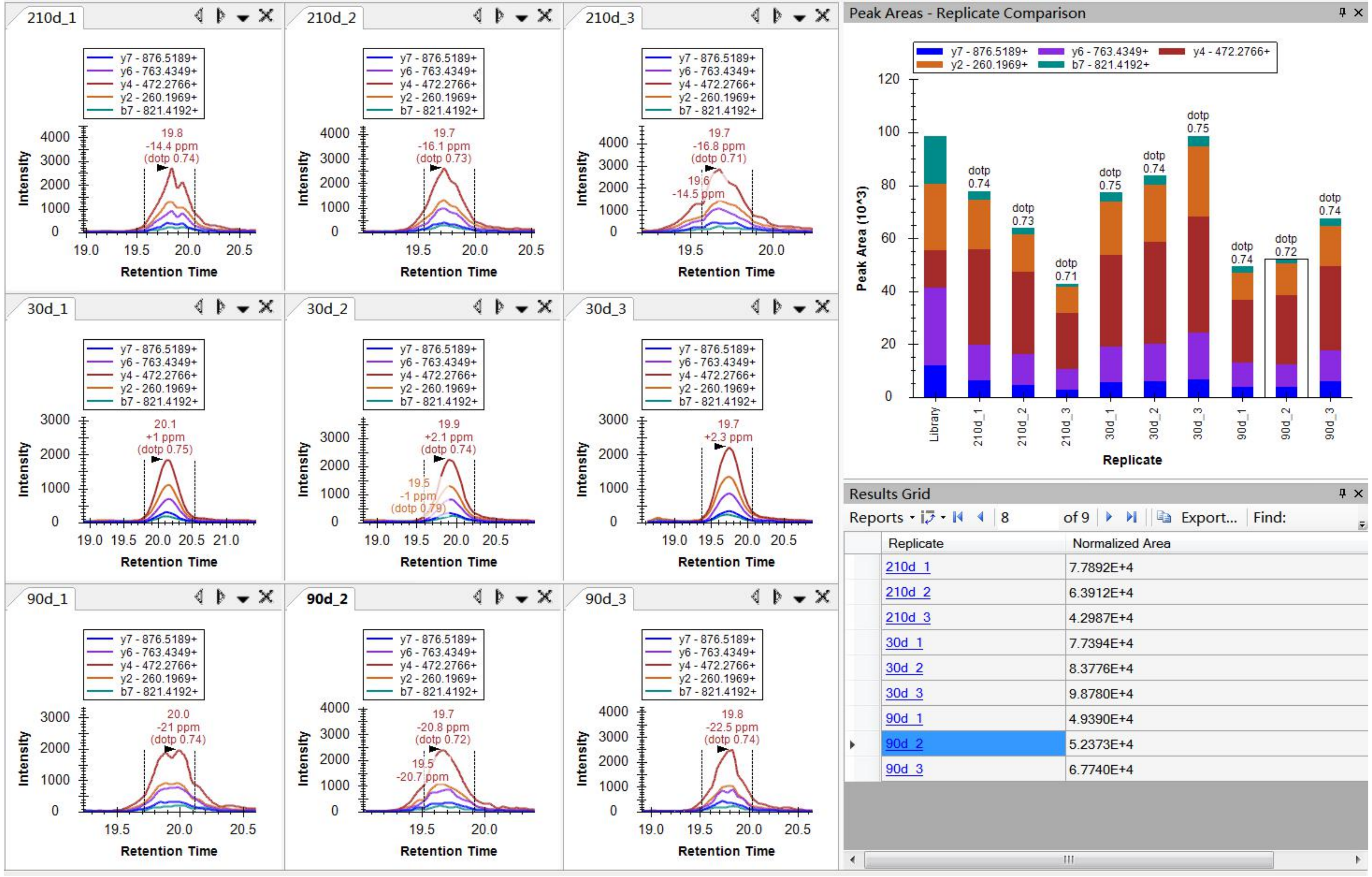

Pig.12644.1(UROD, K.AGLQEAGLAPVPMIIFAK.D [245, 262])

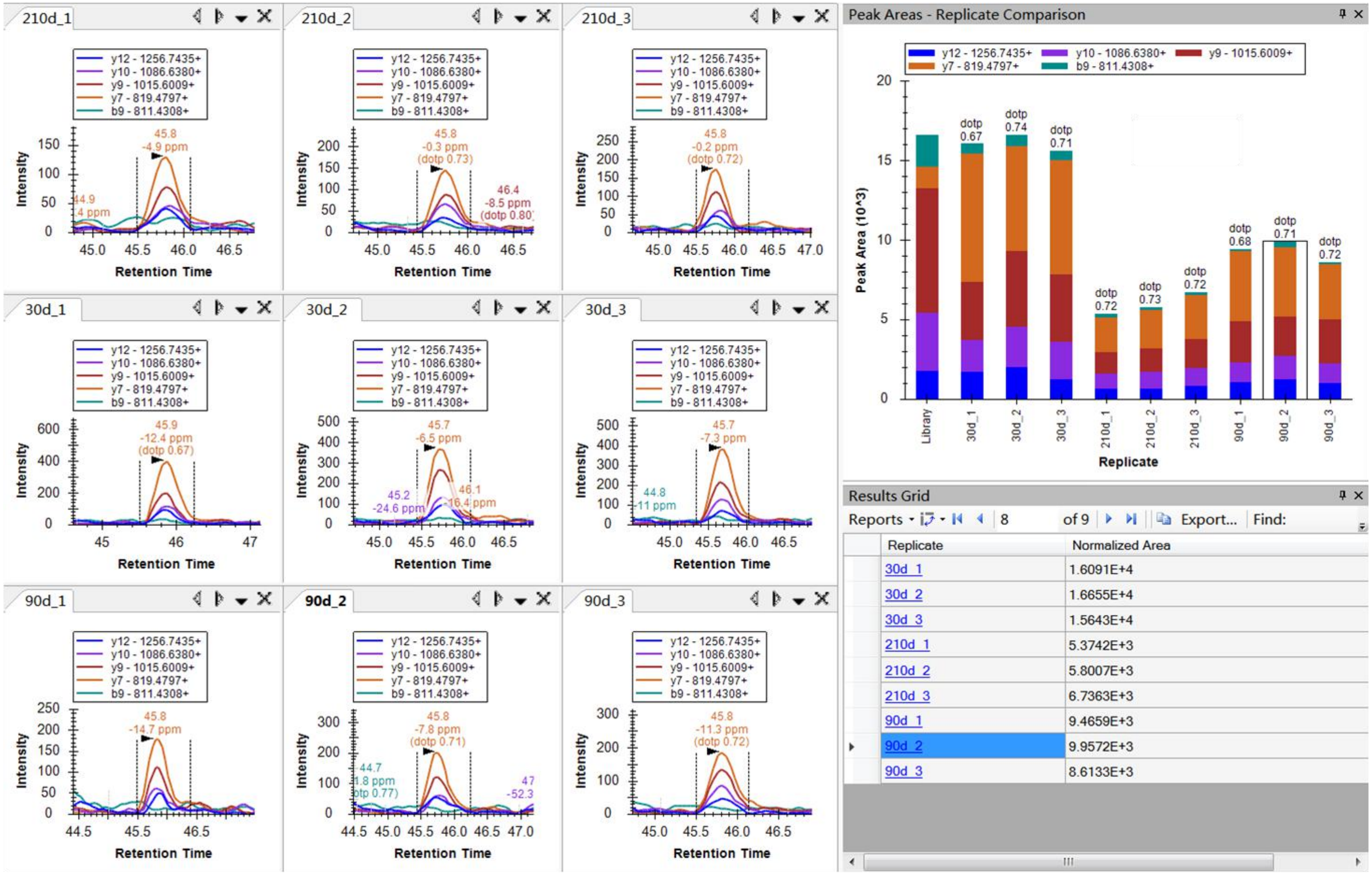

Pig.00413.1(PON2 K.LFVYDPNNPPSSEVLR.I [289, 304])

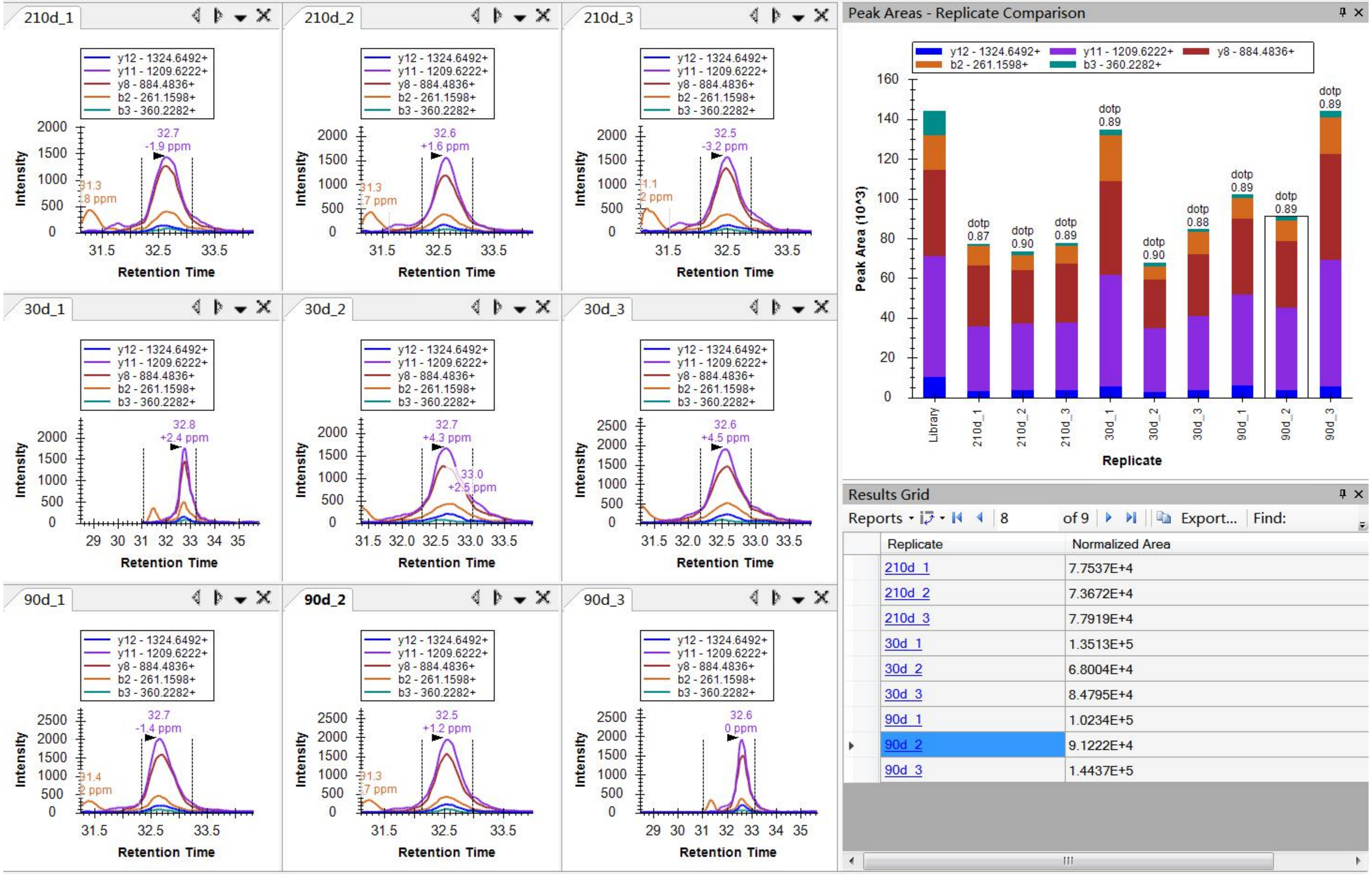

Pig.11729.1(ACADL, K.QGLLGINIAER.H [95, 105])

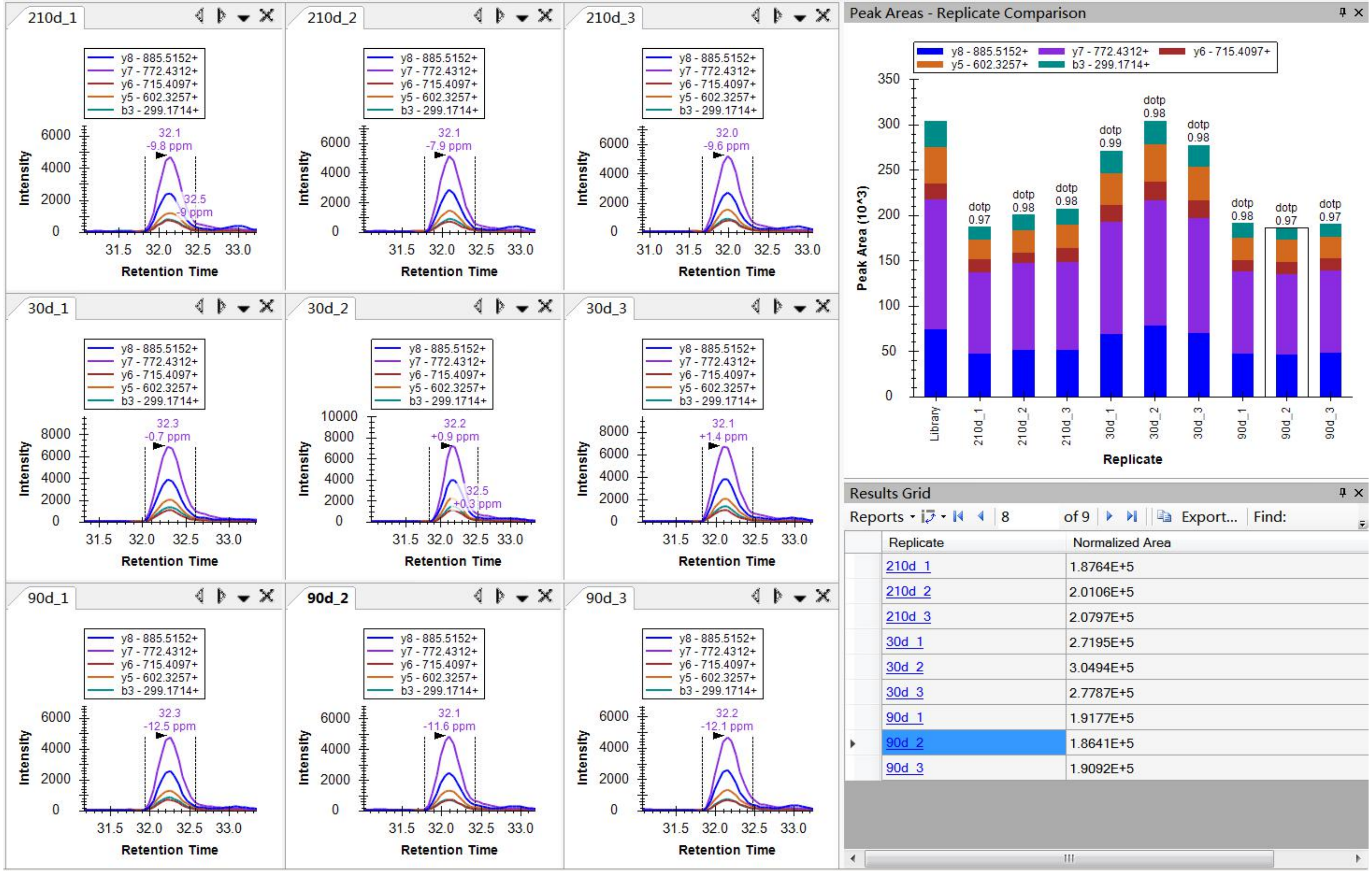

Pig.08197.1(ACAT1, K.LGSIAIQGAIEK.A [65, 76])

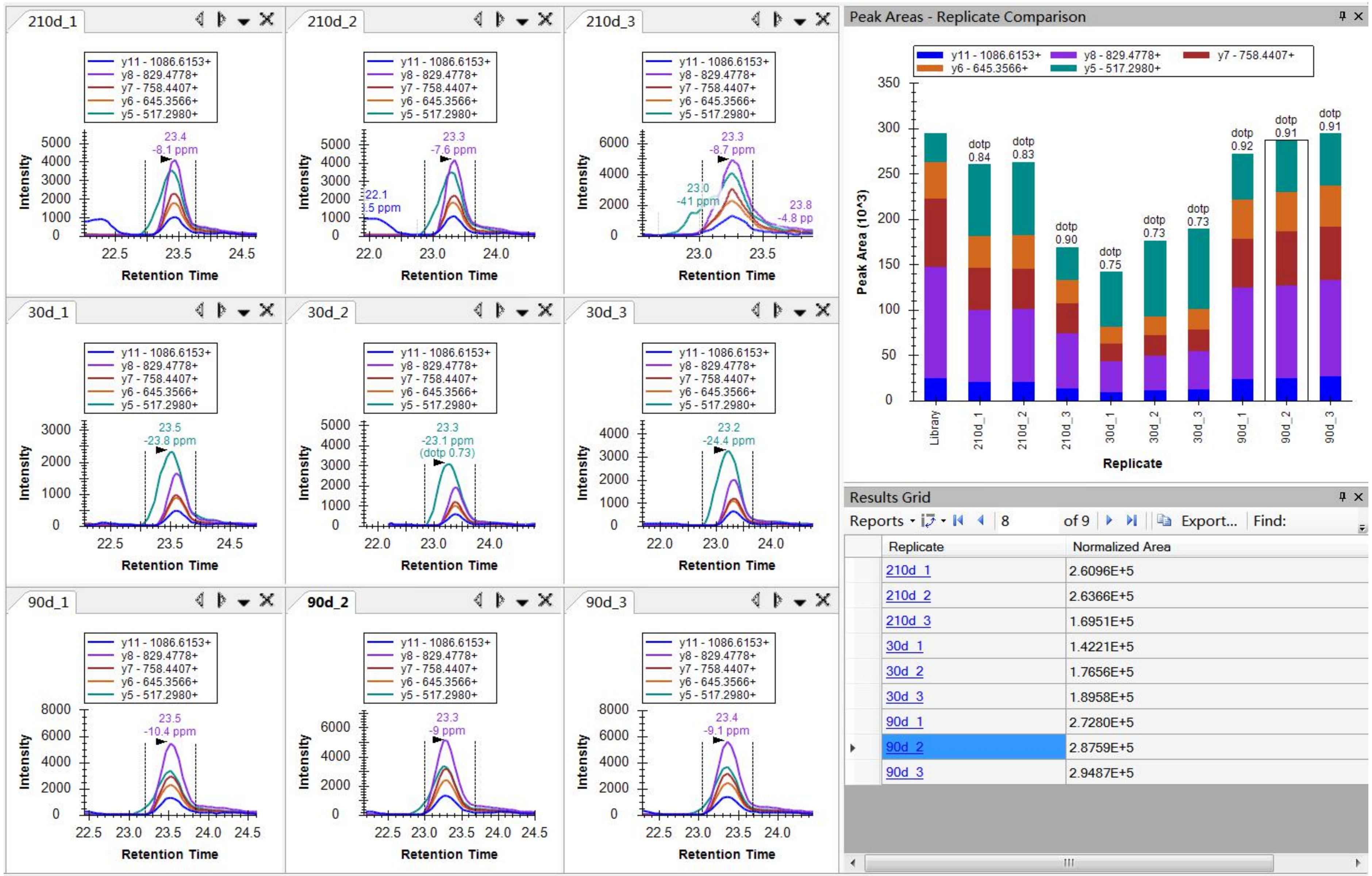

Pig.10018.1(PCK1, R.LTPIGYVPEEAALDLR.G [551, 566])

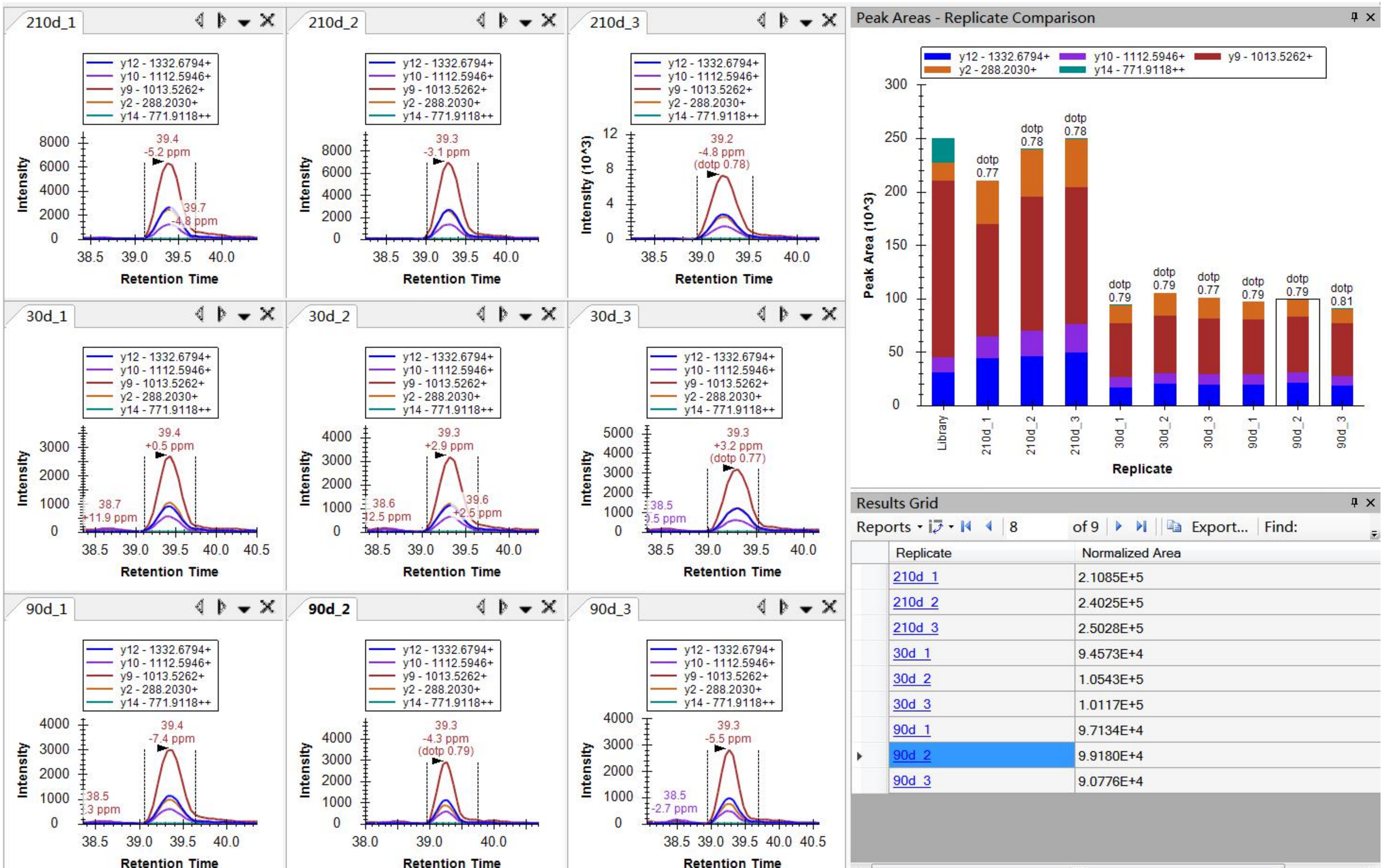

Replicate	Normalized Area
210d_1	2.1085E+5
210d_2	2.4025E+5
210d_3	2.5028E+5
30d_1	9.4573E+4
30d_2	1.0543E+5
30d_3	1.0117E+5
90d_1	9.7134E+4
90d_2	9.9180E+4
90d_3	9.0776E+4

Pig.05906.1(LDHA, R.VIGSGCNLDSAR.F [242, 253])

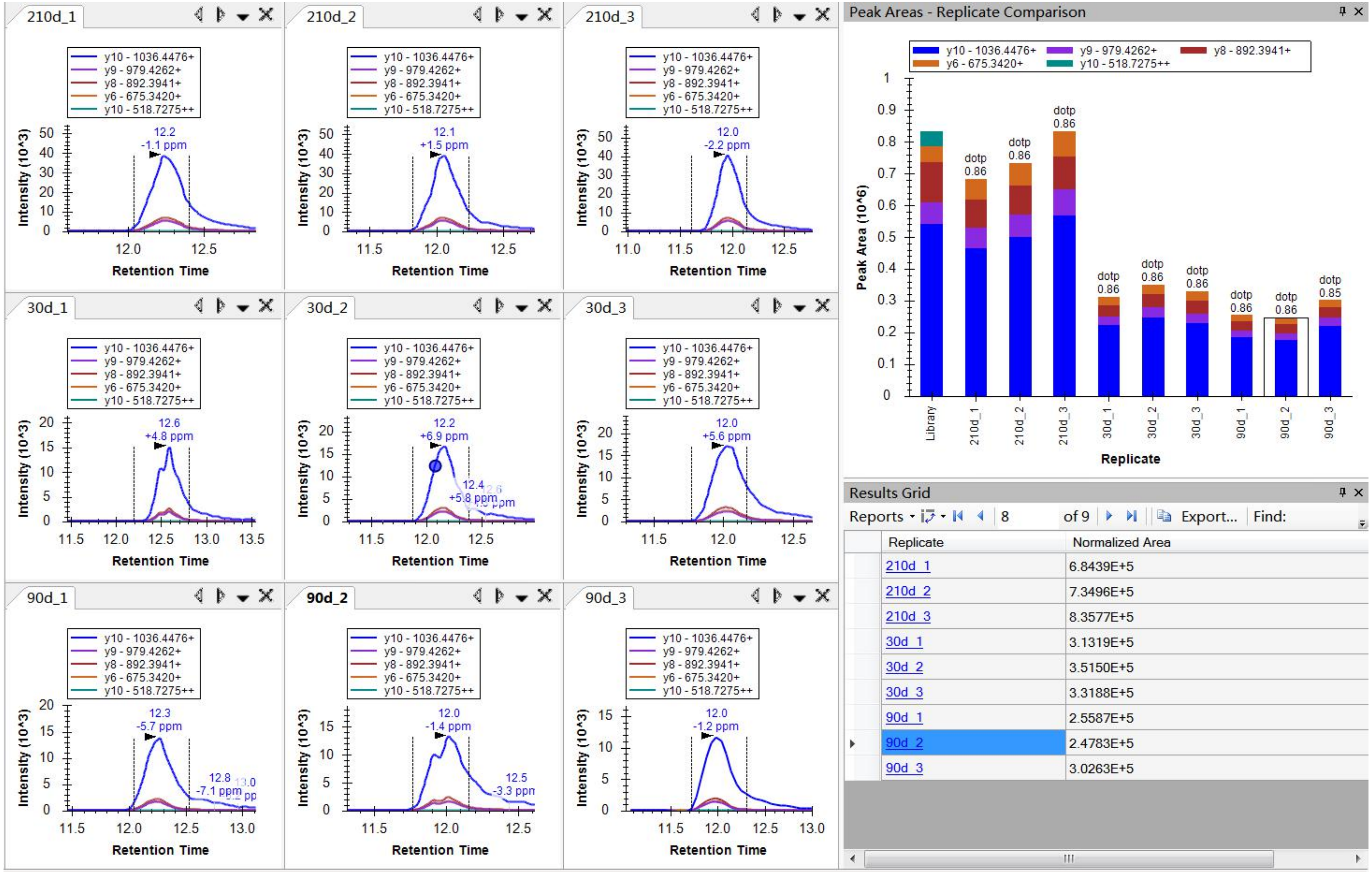

MSTRG.6801 m.6654(K.AYVEANQMLGDLIK.V [892, 905])

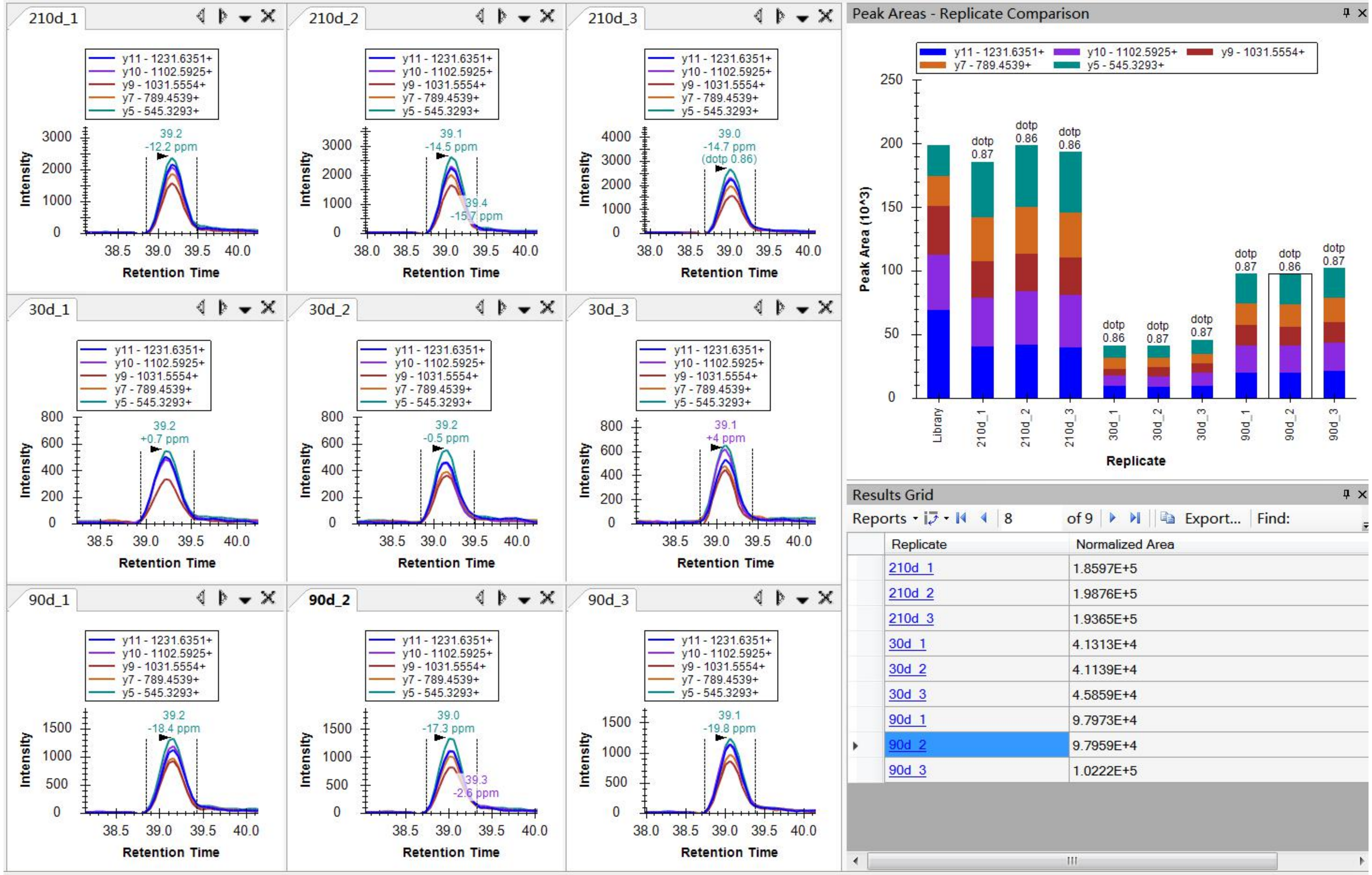

Pig.03993.1(ECHDC1, R.DLLGTVWGGPANLEAVAR.R [276, 293])

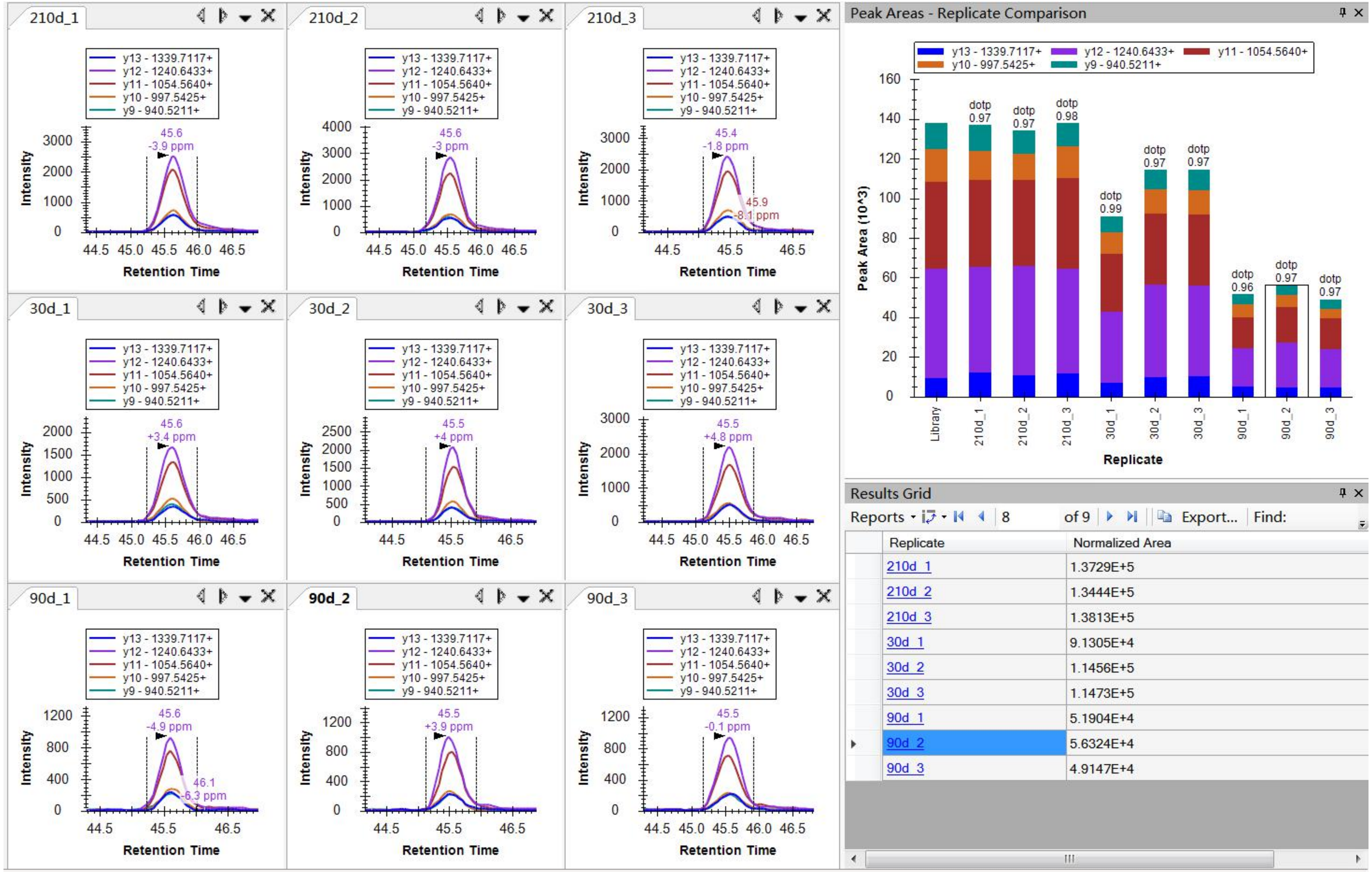

Pig.07780.1(AUH, R.EFLPQGPVAIR.V [225, 235])

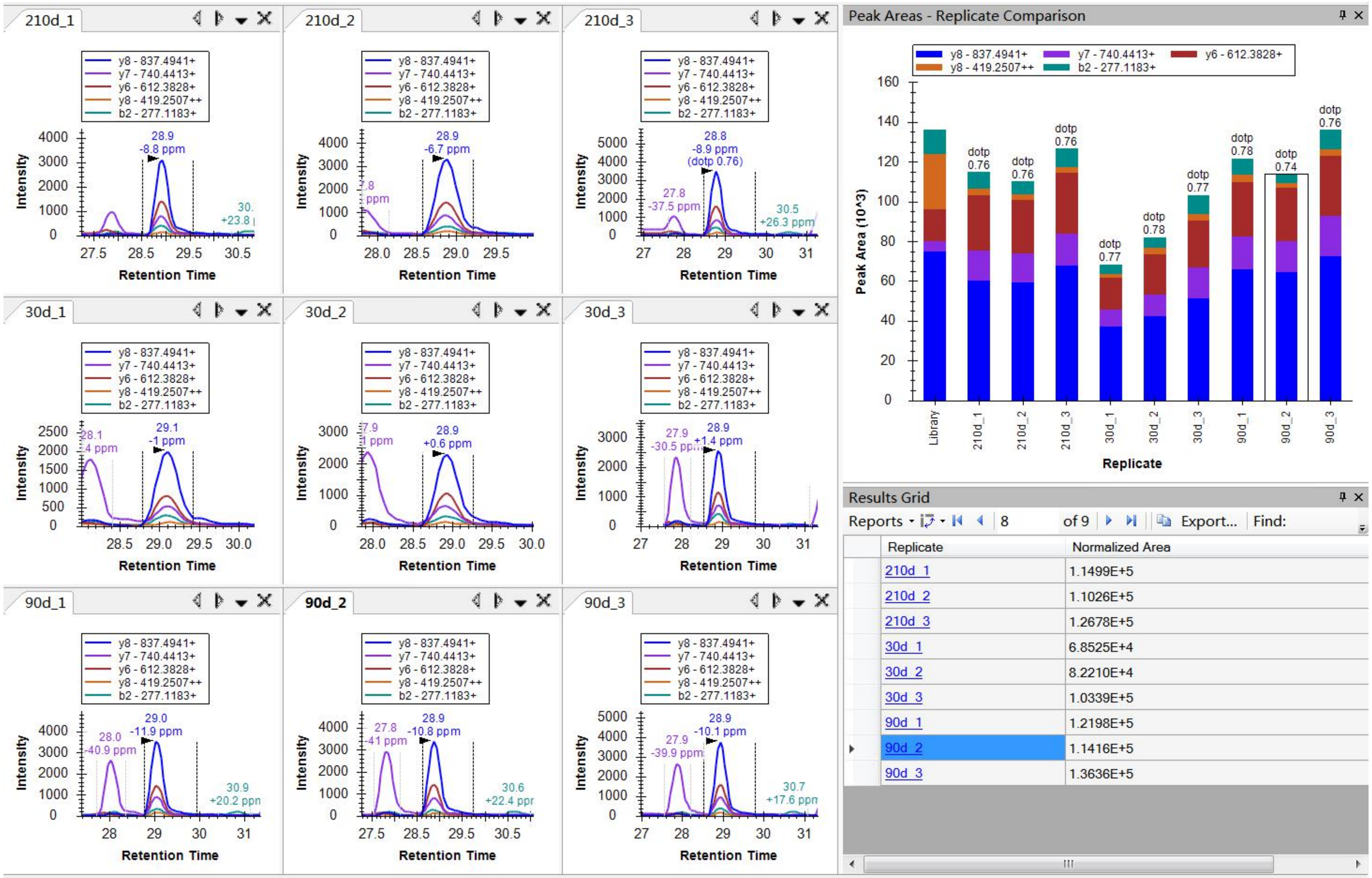

MSTRG.7213_m.7006(K.NFGIGQDIQPK.R [42, 52])

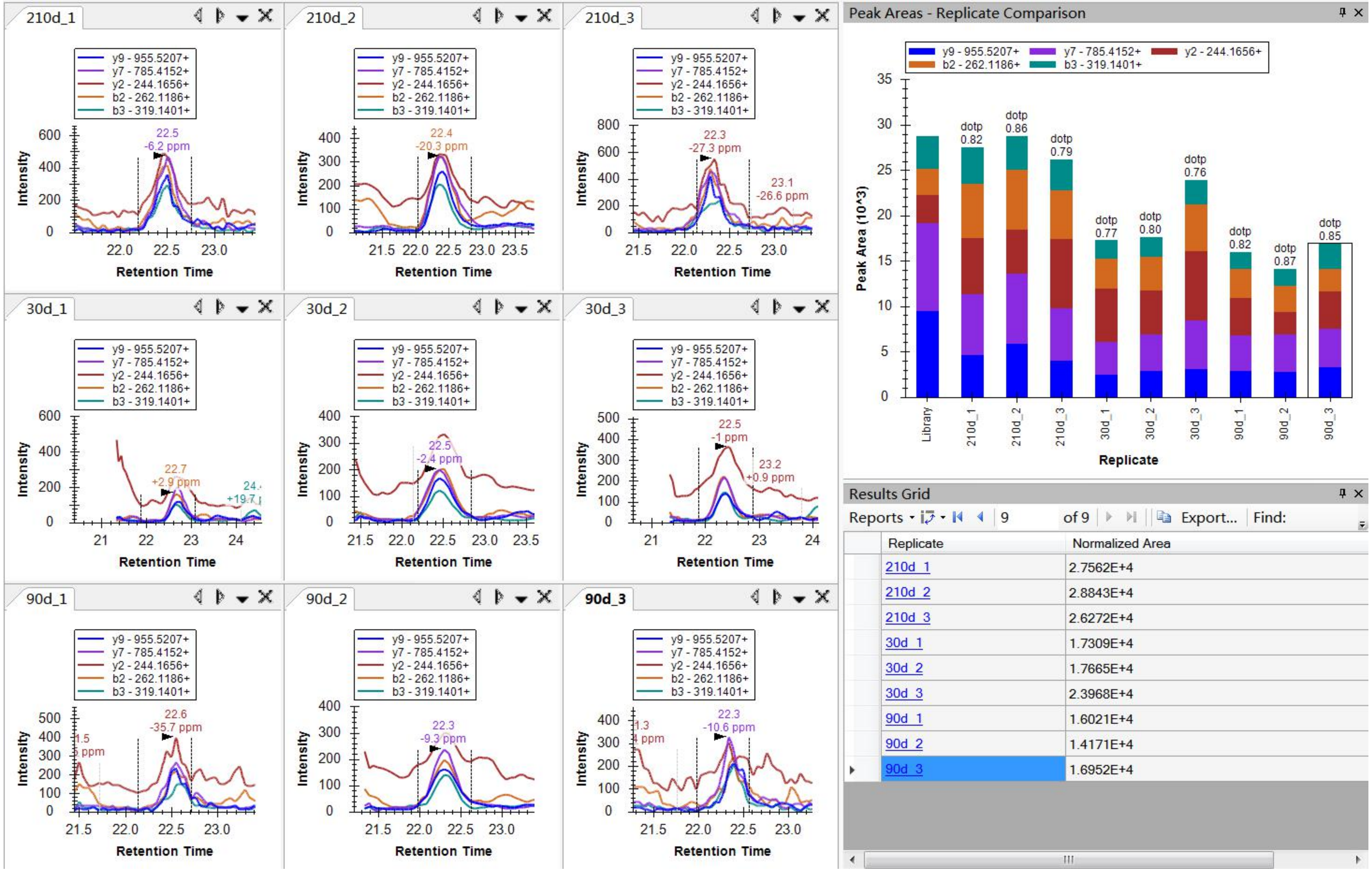

Supplement: Supplementary Figure 4 — Validation of the expression of DEPs by parallel reaction monitoring (PRM). [file Data_Sheet_2.PDF]
